# Superiority and cost-effectiveness of monthly extended-release buprenorphine versus daily standard of care medication: a pragmatic, parallel-group, open-label, multicentre, randomised, controlled, phase 3 trial

**DOI:** 10.1016/j.eclinm.2023.102311

**Published:** 2023-11-17

**Authors:** John Marsden, Mike Kelleher, Eilish Gilvarry, Luke Mitcheson, Jatinder Bisla, Angela Cape, Fiona Cowden, Edward Day, Jonathan Dewhurst, Rachel Evans, Will Hardy, Andrea Hearn, Joanna Kelly, Natalie Lowry, Martin McCusker, Caroline Murphy, Robert Murray, Tracey Myton, Sophie Quarshie, Rob Vanderwaal, April Wareham, Dyfrig Hughes, Zoë Hoare

**Affiliations:** a Addictions Department, School of Academic Psychiatry, Institute of Psychiatry, Psychology and Neuroscience, King’s College London, United Kingdom; b South London and Maudsley NHS Foundation Trust, United Kingdom; c Cumbria, Northumberland, Tyne and Wear NHS Foundation Trust, Newcastle Addictions Service, Newcastle Upon Tyne, United Kingdom; d King’s Clinical Trials Unit, Research Management and Innovation Directorate, King’s College London, United Kingdom; e NHS Tayside and Dundee Health and Social Care Partnership, Scotland, United Kingdom; f Birmingham and Solihull Mental Health, NHS Foundation Trust, Birmingham, United Kingdom; g Addictions Division, Greater Manchester Mental Health NHS Foundation Trust, Manchester, United Kingdom; h School of Health Sciences, Bangor University, Wales, United Kingdom; i Clinic for Health Economics and Medicines Evaluation, Bangor University, Wales, United Kingdom; j Lambeth Service User Council, South London and Maudsley NHS Foundation Trust, United Kingdom; k Patient and Public Involvement and Engagement Representative, United Kingdom

**Keywords:** Opioid use disorder, Standard of care, Extended-release buprenorphine, Effectiveness, Cost-effectiveness

## Abstract

**Background:**

Daily methadone maintenance or buprenorphine treatment is the standard-of-care (SoC) medication for opioid use disorder (OUD). Subcutaneously injected, extended-release buprenorphine (BUP-XR) may be more effective—but there has been no superiority evaluation.

**Methods:**

This pragmatic, parallel-group, open-label, multi-centre, effectiveness superiority randomised, controlled, phase 3 trial was conducted at five National Health Service community-based treatment clinics in England and Scotland. Participants (adults aged ≥ 18 years; all meeting DSM-5 diagnostic criteria for moderate or severe OUD at admission to their current maintenance treatment episode) were randomly assigned (1:1) to receive continued daily SoC (liquid methadone (usual dose range: 60–120 mg) or sublingual/transmucosal buprenorphine (usual dose range: 8–24 mg) for 24 weeks; or monthly BUP-XR (Sublocade;® two injections of 300 mg, then four maintenance injections of 100 mg or 300 mg, with maintenance dose selected by response and preference) for 24 weeks. In the intent-to-treat population (senior statistician blinded to blinded to treatment group allocation), and with a seven-day grace period after randomisation, the primary endpoint was the count of days abstinent from non-medical opioids between days 8–168 (i.e., weeks 2–24; range: 0–161 days). Safety was reported for the intention-to- treat population. Adopting a broad societal perspective inclusive of criminal justice, NHS and personal social service costs, a trial-based cost-utility analysis estimated the Incremental Cost-effectiveness Ratio (ICER) per quality-adjusted life year (QALY) of BUP-XR versus SoC at the National Institute for Health and Care Excellence threshold. The study was registered EudraCT (2018-004460-63) and ClinicalTrials.gov (NCT05164549), and is completed.

**Findings:**

Between Aug 9, 2019 and Nov 2, 2021, 314 participants were randomly allocated to receive SoC (n = 156) or BUP-XR (n = 158). Participants were abstinent from opioids for an adjusted mean of 104.37 days (standard error [SE] 9.89; range: 0–161 days) in the SoC group and an adjusted mean of 123.43 days (SE 4.76; range: 24–161 days) in the BUP-XR group (adjusted incident rate ratio [IRR] 1.18, 95% confidence interval [CI] 1.05–1.33; p-value 0.004). The incidence of any adverse event was higher in the BUP-XR group than the SoC group (128 [81.0%] of 158 participants versus 67 [42.9%] of 156 participants, respectively—most commonly rapidly-resolving (mild–moderate range) pain from drug administration in the BUP-XR group (121 [26.9%] of 450 adverse events). There were 11 serious adverse events (7.0%) in the 158 participants in the BUP-XR group, and 18 serious adverse events (11.5%) in the 156 participants in the SoC group—none judged to be related to study treatment. The BUP-XR treatment group had a mean incremental cost of £1033 (95% central range [CR] −1189 to 3225) and was associated with a mean incremental QALY of 0.02 (95% CR 0.00–0.05), and an ICER of £47,540 (0.37 probability of being cost-effective at the £30,000/QALY gained willingness-to-pay threshold). However, BUP-XR dominated the SoC among participants who were rated more severe at study baseline, and among participants in maintenance treatment for more that 28 days at study enrolment.

**Interpretation:**

Evaluated against the daily oral SoC, monthly BUP-XR is clinically superior, delivering greater abstinence from opioids, and with a comparable safety profile. BUP-XR was not cost-effective in a base case cost-utility analysis using the societal perspective, but it was more effective and less costly (dominant) among participants with more severe OUD, or those whose current treatment episode was longer than 28 days. Further trials are needed to evaluate if BUP-XR is associated with better clinical and health economic outcomes over the longer term.

**Funding:**

Indivior.

**Research in context**

**Evidence before this study**

We searched PubMed and Embase from Jan 1, 2015, to Feb 24, 2023, for publications in English evaluating the clinical efficacy and cost-effectiveness of extended-release buprenorphine for opioid use disorder (OUD). Keywords included ‘injections, subcutaneous/intramuscular/subdermal’; ‘depot’; ‘extended-release/prolonged/sustained-release/long-acting’; ‘monthly/once monthly’. Probuphine®, a 6-monthly subdermal implant, has been evaluated for safety and non-inferiority, but it was discontinued in October 2020. CAM2038 (Buvidal®) is a weekly and monthly subcutaneous injectable product shown to be non-inferior to daily oral buprenorphine. RBP-6000 (Sublocade® and Subutex prolonged release solution for injection [Subutex® PRO]) is a monthly subcutaneous injectable product with a successful double-blind, placebo-controlled evaluation. No effectiveness superiority randomised controlled trials of extended-release buprenorphine (BUP-XR) with standard-of-care (SoC) medications were found. Searches of economic evaluations relevant to the UK identified a health technology assessment by the All-Wales Medicines Strategy Group in which Buvidal® dominated daily buprenorphine/naloxone—but cost savings rested on information medication dispensing costs from clinical expert opinion. A similar evaluation considered by the Scottish Medicines Consortium assumed Buvidal® clinical equivalence with buprenorphine/naloxone in patients for whom oral methadone is not suitable. While Buvidal® was considered cost-saving in the base case, this was reversed when the comparator was changed to buprenorphine.

**Added value of this study**

This open-label, multicentre, phase III randomised controlled trial showed that monthly subcutaneously injected BUP-XR was superior to daily oral SoC in reducing opioid use. There was evidence that BUP-XR was acceptable, and study participants allocated to it stayed in treatment longer, had more likelihood of early OUD remission, experienced either no or reduced opioid craving, and had better patient-reported and clinician-reported outcomes. BUP-XR was more costly than the SoC and associated with greater utility, but it was not cost-effective at a willingness-to-pay threshold of £30,000 per QALY. This challenges the assumption that injectable forms of buprenorphine will necessarily result in cost savings; but it does provide evidence that BUP-XR may improve health related quality of life. BUP-XR also dominated the SoC among participants rated more severe at baseline, and for those in treatment for more than 28 days.

**Implications of all the available evidence**

Evaluated against the daily oral SoC, monthly BUP-XR is clinically superior, delivering greater abstinence from opioids, longer treatment retention, less opioid craving, greater likelihood of early OUD remission, and with a comparable safety profile. The present study was conducted in real-world NHS treatment conditions with participants enrolled at admission or during maintenance treatment. The present study was conducted in real-world NHS treatment conditions with participants enrolled at admission or during maintenance treatment and findings are generalisable in that context. BUP-XR is an evidence-supported treatment option for people who do not want a daily treatment. Further trials are needed to evaluate if BUP-XR is associated with better clinical and health economic outcomes over the longer term.

## Introduction

In 2020, an estimated 61 million people around the world used heroin or non-medical pharmaceutical opioids.^1^ Overdose and accidental poisoning from these drugs can induce a respiratory depression causing fatal cardiorespiratory arrest.^2^ The country-level prevalence of opioid-related mortality varies widely (comparable country rate per million people in 2020 or 2021 was 17 in the EU,^3^ 42 in Australia,^4^ 80 in England and Wales,^5^ 245 in Scotland,^6^ and 292 in the US^7^). OUD can arise and is maintained by hard-to-control craving and compulsive drug taking, despite personal and social harms (DSM-5).^8^ In 2019, 13 million years of healthy life were lost globally to OUD-related premature death and disability.^9^

Daily opioid agonist/partial agonist maintenance treatment is the international standard-of-care (SoC) for OUD. SoC medications are oral (liquid) methadone (MET), a full *μ*-opioid receptor (OR) agonist (usual dose range: 60–120 mg) and buprenorphine (BUP), a partial *μ*-OR agonist and *κ*-OR antagonist, capable of blocking the subjective reinforcing effects of opioids (usual dose range: 8–24 mg).^10^ BUP is formulated in transmucosal products for sublingual or buccal use, including a tablet, a tablet and polymer film combined with naloxone (BUP-NLX), and a lyophilisate wafer (Espranor®)—all forms of BUP are BUP-SL herein unless otherwise stated.

In the UK, primary and secondary care National Health Service (NHS) clinics and non-governmental (third-sector) services provide the OUD SoC. Treatment is initiated by observed daily dosing at community pharmacies. Adherent patients can collect their prescription at set intervals for up to 14 days to take medication at home. During April 2019–March 2022, 71,034 people in England were enrolled in the SoC—the majority with heroin use disorder. A further 69,565 people were enrolled in the SoC with dual OUD and cocaine use disorder (OUD-CUD).^11^ Use of illicit benzodiazepines among the OUD population is also a recognised problem contributing to the risk of fatal overdose.^12^

On average, MET or BUP-SL maintenance is associated with reduced opioid use and increased abstinence^13^^,^^14^; a reduced risk of fatal overdose^15^; and improvements on various dimensions of personal and social functioning.^16^ However, the SoC is sub-optimal for many patients. For example, in a cohort of 12,745 patients in England enrolled for 12–26 weeks in the SoC, 64% were using heroin on ≥10 days in the past month.^17^ In another English cohort of 54,347 patients, just 22% were abstinent when they left treatment.^18^ There is a meta-analysis estimate that 53% of patients enrolled in BUP-SL and 63% enrolled in MET maintenance complete their treatment successfully; and that 50–90% relapse to opioid use one month after discharge.^19^

There can be several reasons why a patient leaves SoC maintenance—but a notable one is that pharmacy dosing is experienced as stigmatising^20^ Some patients do stay in maintenance treatment for many months—but a sizeable minority does not achieve or maintain a clinically significant reduction in their opioid use. For example, in a cohort of 7719 patients in England enrolled continuously in the SoC for over five years, 15% were using heroin on about half of the days each month at each bi-annual assessment.^21^

Extended-release BUP (BUP-XR) may address the effectiveness limitations of the SoC. Two subcutaneous injectable formulations have been developed. Using liquid crystal technology, Camurus developed a weekly and monthly injection (CAM 2038). After non-inferiority evaluation against BUP-SL,^22^ CAM 2038 is now available as Buvidal® in Australia, the EU and the UK. Using biodegradable polymer technology, Indivior developed a monthly injection (RBP-6000). A double-blind, placebo-controlled trial over six months (300 mg each month; or two 300 mg loading doses a month apart followed by monthly maintenance injections of 100 mg) achieved a 24-week abstinence rate in the active and placebo groups of 42.7% and 5.0%, respectively.^23^ RBP-6000 is now available as Sublocade® in several countries including Australia, Canada, Israel, New Zealand, and the US; or as Subutex® PRO in the EU and the UK (UK product license granted on 4 July 2023).

The important question is whether BUP-XR is superior to the SoC for patients at admission and among those enrolled in the SoC who want a convenient and effective alternative. The aim of the **EX**tended-release **P**harmacotherapy for **O**UD (EXPO) study was to determine if BUP-XR is associated with greater reduction in opioid use. We also hypothesised that BUP-XR would be associated with longer time enrolled in study treatment, a greater likelihood of OUD early remission, less opioid craving, and better clinician-reported and patient-reported outcomes. The study included evaluations of safety and cost-effectiveness.

## Methods

### Study design

EXPO was a pragmatic, parallel-group, open-label, multi-centre, phase III, superiority randomised controlled trial of BUP-XR versus MET or BUP-SL (the SoC). The effectiveness endpoint was 24 weeks. The study was registered with EudraCT (2018-004460-63) and ClinicalTrials.gov (NCT05164549) and is completed. Details of the study protocol have been published.^24^

The study was conducted under routine clinical conditions at five NHS community addiction treatment clinics in England (South London [Brixton; with responsibility for study coordination]; West Midlands [Solihull and Wolverhampton]; North-West [Bolton and Salford]; North-East [Newcastle]; and in Scotland (Dundee). Each clinic followed the UK clinical guidelines and offered MET and BUP-SL via assessment and patient preference.

The UK Medicines and Healthcare Product Regulatory Agency approved the study protocol on 4 March 2019. Research materials were approved by the Health Research Authority (IRAS project number: 255522) via the London–Brighton & Sussex Research Ethics Committee (reference: 19/LO/0483) on 14 June 2019.

An independent Steering Committee and Data Monitoring Committee provided study oversight. The King’s Health Partners Clinical Trials Office monitored safety and quality. The King’s Clinical Trials Unit provided an independent web-based randomisation service; designed an electronic data capture system (InferMed MACRO); and produced reports for data verification.

In parallel at the Brixton clinic only, there was random allocation of participants to two additional groups: SoC with personalised psychosocial intervention (PSI) and BUP-XR with PSI. Due to a hiatus in participant recruitment required by the COVID-19 pandemic restrictions, we decided to complete participant recruitment to all groups at the same time. In a protocol amendment, this decision re-framed the PSI study as a mixed-methods evaluation. The EXPO protocol also included a planned analysis of primary outcome effect mediation; nested qualitative and mixed-methods studies of patients’ experiences of BUP-XR at the 24-week endpoint and also for those who opted to received longer-term BUP-XR to the end-of-study; and data-linkage research with UK health and social registry data. Findings from the PSI evaluation and these other studies will be reported elsewhere. The Statistical Analysis Plan (SAP) and Health Economic Analysis Plan (HEAP) were published on the Open Science Framework before data-lock (https://osf.io/qupz8/). Reporting adhered to the CONSORT PRO guideline for pragmatic trials^25^ and the Consolidated Health Economic Evaluation Reporting Standards (CHEERS) guideline for cost-effectiveness evaluations.^26^

### Participants

Participants were adults (≥18 years) with moderate or severe DSM-5 OUD at admission to their current SoC maintenance treatment episode.^27^ All had an allocated clinic keyworker and were offered fortnightly or monthly sessions for medication management and general counselling. Keyworkers and investigators consulted the Electronic Health Record (EHR) and approached patients to discuss the study. There was no recruitment of participants via media advertising. Participants provided their written consent. Study exclusion criteria were defined as clinically significant hypertension; cardiovascular disease; hepatitis or hepatic insufficiency; severe alcohol use disorder; history of allergic/adverse reactions and contraindications to study medication; enrolment in naltrexone (opioid antagonist) relapse prevention treatment in the past three months; uncontrolled mental health disorder; suicide plan or attempt in past six months; and criminal justice involvement that risked incarceration. Study eligible BUP patients were receiving ≤24 mg/day (Espranor® ≤18 mg/day). We initially set a dose threshold of ≤50 mg/day for MET to commence screening with a taper to 20–30 mg/day before conversion to BUP-SL. After a protocol amendment, we decreased the dose threshold for screening initiation to ≤30 mg/day to help participants have time to complete the BUP-SL run-in within seven days. Those patients on higher doses of maintenance medication who wished to take part in the study were offered a taper to the point where they could commence screening.

### Randomisation and masking

The randomisation (no masking of participants) to SoC and BUP-XR (1:1) procedure used random blocks of varying size for even allocation, with stratification by treatment clinic and non-medical drug injecting in the past 28 days (no/yes). Baseline drug injecting status was used because this has been shown to predict negative outcome.^28^

### Procedures

Before their allocation to study treatment, participants completed a face-to-face, investigator-administered interview to record their demographic and treatment characteristics, and to complete baseline clinical assessments, clinician-reported outcomes, a patient-reported measure and patient-reported outcomes.

Clinical assessments were as follows:

*Adult Service Use Schedule* (ADSUS), a structured interview for the past 90 days to record use of hospital services (Accident and Emergency, outpatient, and inpatient care), local authority social services, criminal justice service contacts, and days off work due to illness^29^;
*Alcohol consumption: frequency, quantity, and maximum* (ALC-FQM) to record the number of drinking days in the past 28 days; the typical quantity of alcohol consumed on a drinking day; and the maximum quantity of alcohol consumed on any one day (from the *Treatment Outcomes Profile*)^30^;
*Craving Experience Questionnaire—frequency version* (CEQ-F), an 11-item rating scale recording the frequency of intensity, imagery, and intrusiveness aspects of opioid craving and cocaine craving in the past 14 days (total score range: 0–110; higher score indicating more frequent craving experience)^31^;
*Difficulties in Emotion Regulation*—*Short Form* (DERS-SF), an 18-item scale on emotional awareness, control, and response (total score range: 18–90; higher score indicating more emotion dysregulation)^32^;
*EQ-5D-5L*, a brief generic scale of mobility, self-care, usual activities, pain/discomfort, and anxiety/depression (each dimension scored 1–5 [no problems–extreme problems]) converted into a utility score using the EQ-5D-5L crosswalk to National Institute for Health and Care Excellence (NICE) recommended tariff values for the UK^33^;
*Montreal Cognitive Assessment* (MoCA), version 7.1 at baseline, alternate form version 7.2 at week-12 follow-up included in the protocol for an analysis of treatment effect mediation^34^;
*Structured Clinical Interview for DSM-5 disorders—research version* (SCID-5-RV, 11 symptoms [presence or absence] to diagnose the severity of OUD and CUD [mild: two or three symptoms; moderate: four or five symptoms; severe: six or more symptoms] and applying the definition of ‘early remission’ at 12-week and 24-week follow-up^27^;
*Timeline Follow-back* (TLFB) the field-standard, structured interview adapted to record each day the participant reported using heroin and other non-medical opioids, cocaine, and benzodiazepines at each clinic visit for each day between visits up to a maximum interval of 90 days^35^;
*Urine Drug Screen* (UDS), using the Abbott SureStep™ lateral flow chromatographic immunoassay for qualitative detection of (d-meth)amphetamine, benzodiazepines, BUP, cocaine (benzoylecgonine, cocaine’s unique metabolite), heroin (6-acetylmorphine, heroin’s unique metabolite), morphine, fentanyl and oxycodone and related compounds;
*Visual Analogue Scale*, a single item measure of perceived need (VAS-N) and want (VAS-W) for opioids and for cocaine (maximum strength in past 14-days) on a 100 mm line (rated 0–100; higher score indicating greater strength)^36^;
*Quick Inventory of Depressive Symptomatology—Self-Report* (QUIDS-SR), a 16-item measure of depressive symptoms in the past seven days used for the analysis of treatment effect mediation (total score range: 0–27; higher score indicating more symptoms)^37^;
*Work and Social Adjustment Scale* (WSAS), a five-item scale recording how OUD has impaired work and personal life in the past 14-days used for the analysis of treatment effect mediation (total score range: 0–40; higher score indicating more problems)^38^;

Clinician-reported outcomes were as follows:

*Addiction Dimensions for Assessment and Personalised Treatment* (ADAPT), a 14-item rating scale of OUD severity (three items, score range 0–5; higher scores indicating more OUD severity); concurrent problem complexity relating to health, personality, relationships, risk to self and others, housing, and finance (seven items, score range 0–15; higher scores indicating more problems), and subjective recovery capital relating to motivation, outlook and self-management, social network support, and skills and participation (four items, score range 0–11; higher score indicating more recovery capital)^39^;
*Clinical Global Impression*—Severity and Improvement (CGI-S; CGI-I), adapted single 7-point rating scale of the severity level of opioid-related problems at baseline (extremely mild, very mild, mild, moderate, severe, very severe, extremely severe; score range: 1–7) and improvement in problems (very much improved, much improved, minimally improved, no change, minimally worse, much worse, very much worse; score range: 1–7) at follow-up, respectively^40^;

Patient-reported measure and outcomes were as follows:

*Keyworker Contact Form* (KCF), devised for the study, recording the participant’s recall of the number of brief (15 min) and longer (45 min) conversations with their clinical keyworker in the past month for the economic evaluation;
*Patient Reported Outcome*—*Severity and Improvement* (PRO-S; PRO-I), a single seven-point rating of the severity level of opioid-related problems at baseline and extent of improvement at endpoint, using the same seven-point response categories as the CGI-S and CGI-I^40^;
*Service User Recovery Evaluation* (SURE), a 21-item measure of perceived ‘recovery status’ in the following domains: substance use, material resources, outlook on life, self-care, and relationships (total score range: 21–63; higher score indicating more perceived recovery status)^41^;

Participants attended fortnightly visits to their clinic. The TLFB and UDS were completed at each visit. After baseline, the VAS-N and VAS-W were completed every month. The ADAPT, SCID-5-RV, CGI-S/CGI-I, DERS-SF, KCF, PRO-S/PRO-I, SURE, QUIDS-SR, and WSAS were re-administered at week-4, week-12, and week-24 follow-up. The ADSUS, EQ-5D-5L and SCID-5-RV were re-administered at week-12 and week-24 follow-up. The MoCA was scheduled for re-administration at week-12 follow-up but was dropped because it was unfeasible to administer during the pandemic social restrictions.

The Summary of Product Characteristics was the reference document for SoC medication. The Reference Safety Information for all information pertaining to BUP-XR was the Investigator’s Brochure. Adverse events were reporting continuous during the study.

With regards to study medications, study daily SoC was BUP-SL (tablet [0.4 mg, 2 mg, and 8 mg]; tablet with naloxone [4:1 ratio; 2 mg/0.5 mg, 8 mg/2 mg and 16 mg/4 mg]; and Espranor® (2 mg, 8 mg) and MET (1 mg/1 mL). SoC medication was dispensed initially under observation and then, contingent on satisfactory attendance for dispensing and negative opioid UDS monitoring, it was dispensed by interval for self-administration at home. Participants could request to transition between SoC medications during the study.

Study BUP-XR was Sublocade® (100 mg/0.5 mL and 300 mg/1.5 mL in a prefilled syringe). At randomisation to BUP-XR, participants receiving <8 mg/day BUP-SL had a minimum three-day run-in on 8 mg with their last oral dose administered on the day before their first injection. Participants receiving ≥8 mg BUP-SL had their first injection without delay, with their last oral dose taken on the previous day. Participants receiving MET (≤30 mg/day) were converted to BUP-SL and then received 8–24 mg for three days before first injection.

The protocol specified that the second 300 mg loading dose was given after a minimum of 21 days. The four subsequent maintenance injections were scheduled every four weeks (dosing windows in Appendix I, Supplementary Table S1.1, page 2), but each dose could be given up to a maximum of 60 days after the previous one. After this BUP-XR was taken to be discontinued, and any further treatment was initiated with the SoC. During maintenance, if the participant experienced opioid withdrawal symptoms, bothersome craving, or used opioids—and there were no safety concerns—their dose could increase from 100 to 300 mg. It could then stay at that level or reduce to 100 mg on patient request. Supplemental BUP-SL dosing was available as needed between injections to manage withdrawal symptoms.

BUP-XR was administered by subcutaneous injection into abdominal adipose tissue by trained site investigators. Rotating around the quadrants of the transpyloric and transtubercular planes, the needle insertion point was selected with adequate amounts of tissue in a location unlikely to be rubbed or compressed by clothing while avoiding brawny or fibrous tissue or areas with excessive pigment, nodules, lesions, or hair. If there were no safety concerns, participants in the BUP-XR group were able to receive injections over the longer-term to a maximum of 33 months (end-of-study).

### Outcomes

Combining TLFB and UDS data, the primary outcome measure was days of abstinence from all non-medical opioids. After randomisation, this outcome was counted from day 8–168 (i.e., week 2–24; range: 0–161 days). A 24-week effectiveness endpoint is commonly used in intervention trials in the field, and here there was alignment with the pivotal efficacy trial of monthly RBP-6000.^23^ Given the daily versus monthly dosing regimen, a seven-day ‘grace period’ was used to co-ordinate the first injection, so that the count of abstinent days would start at the same time in both treatment groups. The UDS result overrode self-report. With a three-day detection sensitivity, a positive opioid test was recorded on the TLFB record as an opioid-using day on the day of the test and also for the two preceding days.

Secondary outcomes were: (1) treatment retention (number of days enrolled in study treatment and time to any discontinuation of study treatment); (2) OUD and CUD remission status at the study endpoint (in error, the craving item was not discounted for CUD remission status); (3) use of cocaine and benzodiazepines as measured by TLFB and UDS and defined in the same way as the primary outcome); (4) longest duration of continuous abstinence from opioids, cocaine and benzodiazepines; (5) alcohol use (ALC-FQM); (5) endpoint scores on the ADAPT, CEQ-F, VAS-N and VAS-W, CGI-I, DERS-SF, PRO-I, SURE, QUIDS-SR and WSAS measures.

### Statistical analysis

The sample size was estimated for the primary outcome. Informed by a pragmatic effectiveness trial of non-response to the SoC,^42^ with a baseline rate of opioid use (0.6) and a 23% treatment effect, 304 participants were required for the superiority comparison to give 90% power, with alpha at 5%, and with a 15% inflation in recruitment to offset attrition.

The SAP was implemented in STATA (version 16.1) and R (version 1.4.1) and followed the intention-to-treat principle. The Senior Statistician (ZH) was blinded to treatment group allocation. Statistical tests were two-sided and performed using a 5% significance level (reporting 95% confidence intervals [CI]), and the p-value of the effect. There was no adjustment for multiple comparisons.

### Missing data

If available, missing TLFB data for the primary outcome were obtained from available Treatment Outcomes Profile data on the EHR. The EHR was used to capture SoC time in treatment, but this did not include a measure of self-reported SoC adherence. For participants who were not retained to the endpoint and who had more than 5% missing episodic TLFB data, a maximum likelihood multiple imputation approach was planned with predicted mean matching (Stata command: mi impute pmm) using a model with treatment group, the stratification variables, and also baseline variables that predicted missing data. For participants not retained to endpoint, and those with episodic missing TLFB data less than 5% of the total, an offset (censoring) exposure was planned to represent the total amount of missing data for the participant. Mean score substitution was used for missing continuous outcome measures if the level of missing data was <20%, otherwise multiple imputation with predictive mean matching was used, as above.

### Primary outcome analysis

Pooling all data from the five clinics, the primary outcome was modelled by mixed-effects negative binomial regression with fixed effects for treatment group and the drug injecting status stratifier, and a random varying intercept for treatment clinic. The study protocol indicated that participant sex and age would be additionally included in the analysis models; but after review we judged could risk overfitting so these covariables were removed from the SAP before its publication.

The treatment effect parameter was the adjusted number of abstinent days (standard error [SE]) and incident rate ratio (IRR; 95% CI). The unadjusted longitudinal course of opioid use for each participant was plotted in R (command: ggplot2 geom_tile).

There were six planned sensitivity analyses for the primary outcome model: (1) data during the one-week grace period were included (analysis period: day 0–168); (2) the last 14 days were excluded from the analysis (analysis period: day 8–154); (3) the number of days of the current treatment episode preceding study enrolment was included as a covariable; (4) all baseline predictors of missing data were included; (5) a missing data ‘best case’ scenario (i.e., all missing TLFB and UDS data indicating abstinence from opioids); and (6) and a missing data ‘worst case’ scenario (i.e., all missing TLFB and UDS data indicating opioid use). It was originally intended to include the medication preference factor as a sensitivity analysis to the main analysis. However, it was stipulated this would only be conducted if there was an adequate sample of the levels of the preference factor (stated as 50–60%). Only 5% of the sample indicated a preference for MET over BUP therefore this sensitivity analysis was not undertaken.

There were five planned exploratory evaluations of the primary outcome model with the inclusion of the following baseline sub-groups: (1) classification of opioid-related problems as ‘extremely mild–mild’ versus ‘moderate–extremely severe’ on the CGI-S; (2) use of cocaine on ≥1 days in the past 28 days; (2) use of benzodiazepines on ≥1 days in the past 28 days; (3) time in maintenance treatment at the point of study enrolment (<28 days versus ≥28 days); and (5) the addition of a COVID-19 analysis to the SAP to create a grouping variable to indicate if participant enrolment was before the first clinic paused study enrolment due to the pandemic restrictions (20 March 2020) versus participant enrolment after the first study clinic had resumed study enrolment (12 June 2020). Additionally, a planned per protocol analysis would be conducted if the occurrence of protocol deviations rose above 10%.

### Secondary outcome analysis

The analysis of the number of days from randomisation to (any) study discontinuation—with the proportional hazard assumption assessed via the interaction of treatment group and time with censoring of participants on their last day of treatment or at the endpoint if they were continuously enrolled—was by shared frailty Cox regression with fixed effects for treatment group and drug injecting status and a random varying intercept for treatment clinic. Continuous (scale) outcome measures were analysed by a mixed-effects generalised linear model (according to the distribution with a log link), and included treatment group, drug injecting status, and the baseline score of the measure (all fixed effects) and a random varying intercept for treatment clinic.

### Cost-effectiveness analysis

The base-case economic analysis was conducted from a societal cost perspective and, using patient-level data, was a within-trial, intention-to-treat, cost-utility analysis to compare BUP-XR with SoC over the 24 weeks of study treatment. The choice of perspective reflected the likely incurrence of costs beyond the NHS and personal social services (e.g., criminal justice). The analysis was conducted in R (version 4.3.1).

All resource use was measured irrespective of whether it was related to OUD.^43^ All costs were valued in pounds sterling (£) for 2020–2021 and were not discounted as the study follow-up was less than one-year (Appendix II, Supplementary Table S2.1, page 10 shows unit costs and their sources). Total costs for resource use were calculated as the sum-product of unit cost and the recorded number of times that each resource use frequency. The unit costs of BUP and MET and concomitant medicines were taken from the British National Formulary (Appendix II, Supplementary Table S2.2, page 13) to reflect the prices that would be paid by the NHS as closely as possible. The manufacturer provided an indicative unit cost of £262.90 per BUP-XR for the primary analysis, and a lower price of £239.70 to use in cost sensitivity analyses.

Missing cost and utility data were imputed using multiple imputation with chained equations using treatment group, participant’s last visit, and data collected at previous time points (including baseline and randomisation data) as predictors. Measured variables were imputed using predictive mean matching, and variables derived from measured variables (e.g., QALYs, total trial costs) were imputed using passive imputation, to retain the relationships between the variable of interest and those it is derived from. There was one exception to this—criminal justice costs—which were aggregated by timepoint prior to imputation due to issues of model convergence when included separately. Imputation procedures were nested within each bootstrap; consequently, we used one imputation per bootstrap was used instead of basing the number of imputations on the fraction of missing information as the latter may underestimate uncertainty.^44^

The following assumptions were made: (1) SoC treatment was initiated by a nurse prescriber (AfC Band 8a grade) through a 60-min assessment and 45-min follow-up in the first week (to complete dose titration, clinical team and pharmacy discussion, and write up notes); (2) for each complete month of SoC treatment, there was one 15-min session with a nurse prescriber to check and adjust the treatment dose; (3) based on a survey of each clinic, for each 14-day period, patients would collect their SoC medication 8.8 times and 49.6% of those doses were observed—this was tested in two scenario analyses of different dispensing arrangements during treatment, whereby medication was also assumed to have been dispensed 6 times per week with each dose observed; or that there was a single collection each fortnight with no observed dosing; (4) each BUP-XR injection was administered by a nurse prescriber (AfC Band 8a grade) in 16 min (15 min required for the product to reach room temperature once removed from refrigerated storage and 1 min for injection).

Quality-adjusted life years (QALYs) were calculated for each participant using the trapezoidal rule for area under the curve of utilities, derived from EQ-5D-5L responses and NICE’s recommended tariff values.^45^^,^^46^ Both costs and QALYs were modelled with mixed-effects generalised linear models (Gamma distribution with a log link and Gaussian distribution with an identity link, respectively). Treatment group was included as a fixed effect, clinic and injecting status were included as random intercepts, and baseline costs (or QALYs) included as covariables in the cost (and QALY) regressions, respectively, to control for any imbalances at baseline and to improve precision.^47^ The distribution and link functions were chosen from a set of candidate models (Gamma, Gaussian, or Poisson distribution with log or identity link) based on ability to run with bootstrapped and imputed data, Akaike Information Criterion (AIC) values, and inspection of residual error plots.

The primary economic outcome was the Incremental Cost-effectiveness Ratio (ICER),^48^ calculated as the difference in mean total costs between the intervention groups divided by the respective difference in mean QALYs between the intervention groups. Incremental Net Monetary Benefits (INMB) were also calculated at the £20,000 and £30,000 per QALY thresholds, corresponding to willingness-to-pay thresholds (WTP) normally considered by NICE.^49^ The cost-effectiveness threshold aims to represent the marginal value of health by reflecting opportunity costs in terms of forgone health benefits. When expressed as a cost per QALY, the threshold indicates a health service’s willing to pay for an additional QALY.^50^ Analyses were bootstrapped, with bias-corrected 95% central range (CR) calculated from 10,000 replications for the primary analysis and 5000 replications for all others.

As the cost of OUD treatment varies by patient, delivery and medication cost—and because the price of BUP had varied greatly in the past three years (Appendix II, Supplementary Fig. S2.1, page 15) —we conducted scenario analyses to account for treatment delivery options and medication price, as well as the economic perspective. We also investigated whether cost-effectiveness varied by the following baseline sub-groups: cocaine use; benzodiazepine use; <1 month and ≥1 month in current treatment; and for participants with rated severity on the CGI-S of ‘extremely mild–mild’ or ‘moderate–extremely severe’.

### Role of the funding source

The funder of the study had no role in study design, data collection, data analysis, data interpretation, or writing of the report. JB, ZH, RE, DH and JM had access to the dataset and JM had final responsibility for the decision to submit for publication.

## Results

**Fig. 1:**
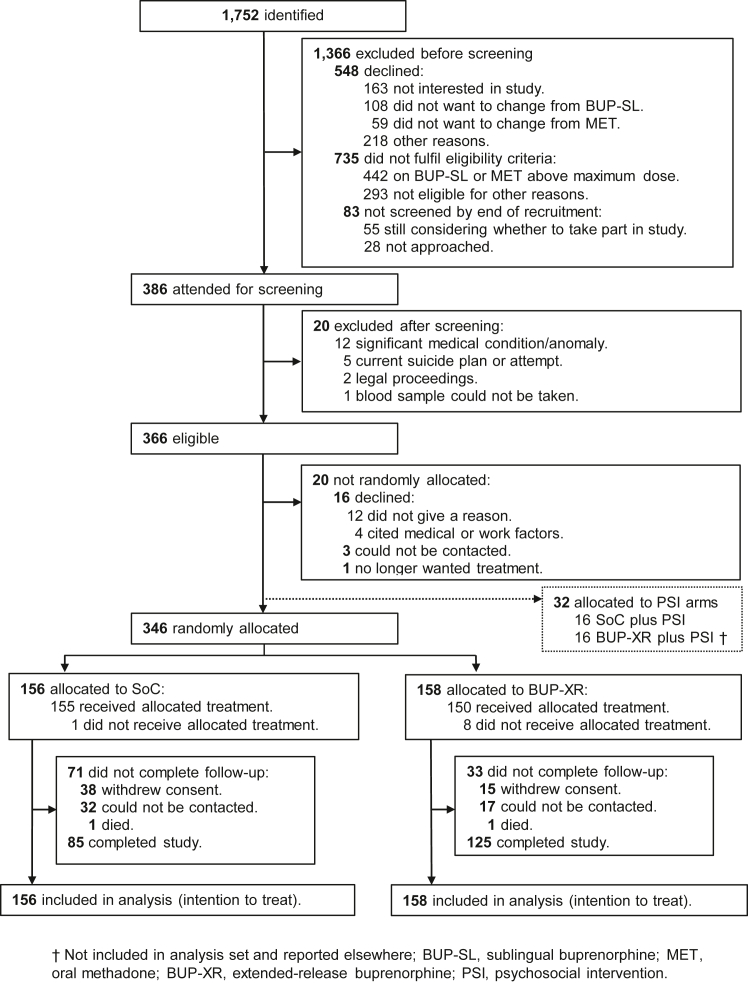
Trial profile.

Between 6 August 2019 and 2 November 2021, we identified 1752 potentially eligible patients of which 1366 patients were excluded before screening—the most common reason was refusal to join the study or SoC dose not within range (study flow in Fig. 1). A total of 314 participants (forming the intention-to-treat population) completed their BUP-SL run-in and were randomly allocated to SoC (n = 156) or BUP-XR (n = 158); 386 participants consented for screening and 366 completed it. For the single-centre trial of medication and PSI, an additional 32 participants completed their BUP-SL and randomly allocated to the SoC plus PSI and BUP-XR plus PSI groups (no further description herein).

The last follow-up visit was on 29 April 2022. Participants in the treatment groups were comparable on demographic and clinical characteristics at baseline (Table 1). Most commonly, OUD related to heroin (285 [90.8%] of 314 participants). The majority of study participants (231 [73.6%] of 314 participants) were in SoC maintenance at study enrolment for an average of 49.9 weeks (95% CI 3.0–176.6).

**Table 1:** Baseline characteristics of the full analysis set.

Characteristic	SoC (n = 156)	BUP-XR (n = 158)	Overall (n = 314)
**Clinic—participants**^a^			
London	46 (29.5)	47 (29.8)	93 (29.6)
Manchester	9 (5.8)	11 (7.0)	20 (6.4%)
Newcastle	51 (32.7)	52 (32.9)	103 (32.8)
West Midlands	24 (15.4)	23 (14.6)	47 (15.0)
Tayside	26 (16.7)	25 (15.8)	51 (16.2)
**Participant—demographic characteristics**			
Age—years	41.6 (8.2)	42.5 (7.9)	42.0 (8.1)
Sex—at birth			
Male	111 (71.2)	122 (77.2)	233 (74.2)
Female	45 (28.9)	36 (22.8)	81 (25.8)
Ethnicity			
White	127 (81.4)	136 (86.1)	263 (83.8)
Black	13 (8.3)	12 (7.6)	25 (7.9)
Mixed	10 (6.4)	7 (4.4)	17 (5.4)
Asian	4 (2.6)	1 (0.6)	5 (1.6)
Other	2 (1.3)	2 (1.3)	4 (1.3)
**Participant—clinical characteristics**			
Age (years) first received SoC (a)	29.8 (9.3)	30.4 (8.8)	30.1 (9.0)
Duration of current SoC episode—mean weeks	39.8 (2.4–167.3)	58.9 (4.0–183.3)	49.9 (3.0–176.6)
New admission for SoC	44 (28.2)	39 (24.6)	83 (26.4)
OUD medication at start of screening (b)			
BUP and BUP-NLX	90 (57.7)	82 (51.9)	172 (54.8)
Dose—mg (mean [SD]; range)	13.5 [5.3]; 4–26^b^	12.7 [5.3]; 2–24	13.1 [5.3]; 2–26
Espranor®	3 (1.9)	9 (5.7)	12 (3.8)
Dose—mg (mean [SD]; range)	15.3 [3.1]; 12–18	15.8 [2.9]; 10–18	15.7 [2.8]; 10–18
MET	15.0 (9.6)	22 (13.9)	37 (11.8)
Dose—mg (mean [SD]; range)	29.5 [9.3]; 20–50	22.2 [7.7]; 4–30	25.2 [9.0]; 4–50
**Clinical assessments**			
**OUD—drug type**			
Heroin	142 (91.0)	143 (90.5)	285 (90.8)
Illicit/non-medical pharmaceutical opioid	14 (9.0)	15 (9.5)	29 (9.2)
**DSM-5 OUD status**			
Moderate	5 (3.2)	1 (0.6)	6 (1.9)
Severe	151 (96.8)	157 (99.4)	308 (98.1)
**DSM-5 CUD status** (c)			
Missing	17 (10.9)	23 (14.6)	40 (12.7)
No symptoms	30 (19.2)	29 (18.4)	59 (18.8)
One symptom	4 (2.6)	9 (5.7)	13 (4.1)
Mild	9 (5.8)	10 (6.3)	19 (6.1)
Moderate	14 (9.0)	13 (8.2)	27 (8.6)
Severe	82 (52.6)	74 (46.8)	156 (49.7)
**Drug use**—past 28 days			
Injecting^a^	17 (10.9%)	12 (7.6%)	29 (9.2%)
Opioids	74 (47.4%)	64 (40.5%)	138 (44.0%)
Cocaine	80 (51.3%)	73 (46.2%)	153 (48.7%)
Benzodiazepines	38 (24.4%)	34 (21.5%)	72 (22.9%)

Characteristic	SoC (n = 156)	BUP-XR (n = 158)	Overall (n = 314)
**Clinical assessments–continued**			
**Alcohol use—past 28 days** (a)			
Use of alcohol—participants	89 (57.1)	86 (54.4)	175 (55.7)
Number of drinking days—mean^c^	5.3 (8.6)	5.1 (8.7)	5.2 (8.6)
Drinks per drinking day—mean^c^	9.3 (8.9)	8.5 (8.3)	8.9 (8.6)
Maximum drinks on drinking day—mean^c^	14.3 (14.0)	13.4 (14.0)	13.9 (14.0)
**Craving for opioids** (a)			
CEQ-F	29.9 (34.3)	27.8 (33.1)	28.8 (33.8)
VAS-N	27.4 (39.4)	25.8 (38.4)	26.6 (38.8)
VAS-W	33.5 (38.9)	31.0 (36.3)	32.2 (37.6)
**Craving for cocaine** (c)			
CEQ-F	26.8 (34.9)	26.5 (34.2)	26.6 (34.5)
VAS-N	18.6 (32.9)	15.3 (29.3)	16.9 (31.1)
VAS-W	29.0 (37.6)	27.6 (35.7)	28.3 (36.6)
**DERS**—mean (d)	44.8 (15.3)	44.4 (15.2)	44.6 (15.2)
**QUIDS-SR**—mean (a)	8.5 (4.9)	8.5 (4.8)	8.5 (4.8)
**WSAS**—mean (d)	17.0 (12.8)	16.1 (13.6)	16.5 (13.2)
**Clinician-reported outcomes**			
**ADAPT**			
OUD addiction severity—mean	2.1 (1.7)	1.9 (1.6)	2.0 (1.7)
Concurrent problem complexity—mean	3.1 (2.4)	3.1 (2.7)	3.1 (2.5)
Recovery strengths—mean	7.0 (2.1)	7.1 (2.2)	7.0 (2.1)
**GSI-S—severity of opioid-related problems**			
Extremely mild–mild	90 (57.7)	90 (57.0)	180 (57.3)
Moderate–extreme	66 (42.3)	68 (43.0)	134 (42.7)
**Patient-reported outcomes**			
PRO-S—level of opioid-related problems (a)			
Less than moderate—participants	103 (66.0)	99 (62.7)	202 (64.3)
Moderate or more—participants	53 (34.0)	47 (36.7)	111 (35.4)

Data are n (%); mean (SD).

Missing data is: (a) 1 participant in the BUP-XR group; (b) 4 participants in the SoC group and 6 participants in the BUP-XR group; (c) 17 participants in the SoC group and 23 participants in the BUP-XR group; (d) 1 participant in the SoC group and 1 participant in BUP-XR group.

ADAPT, Addiction Dimensions for Assessment and Personalised Treatment; BUP-XR, extended-release buprenorphine; CUD, cocaine use disorder; CEQ-F, Craving Experiences Questionnaire-frequency version; DERS-SF, Difficulties in Emotion Regulation Scale-Short Form; GSI-S, Global Severity Index-Severity; OUD, opioid use disorder; VAS-N, visual analogue scale of perceived need for drug; VAS-W, visual analogue scale of perceived want for drug; PRO-S, patient rated evaluation of severity; SoC, standard-of-care; QIDS-SR, Quick Inventory of Depressive Symptomatology-Self-Report; WSAS, Work and Social Adjustment Scale.

a Stratification variable.

b Single case deviation, one participant commenced screening at 26 mg/day (2 mg/day above protocol) and was managed in sensitivity analysis.

c UK standard unit contains 8 g ethanol.

As defined by attendance for the endpoint clinic interview, 210 (66.9%) of the 314 participants in the full-analysis set completed the study (85 [54.5%] of 156 participants in the SoC group and 125 [79.1%] of 158 participants in the BUP-XR group). The study completion rate was reflected in a greater likelihood of withdrawal of consent in the SoC group than the BUP-XR group (38 [24.4%] of 156 versus 15 [9.5%] of 158, respectively).

During a data review, we identified that 21 [13.5%] of 156 participants in the SoC group and 25 [15.8%] of 158 participants in the BUP-XR group attended their endpoint follow-up slightly earlier than scheduled. This was usually only 1–2 days before, but it meant that the TLFB and UDS data for these 46 participants fell short of 161 days. Available data from the I was used to complete the dataset. Eighteen [5.7%] of 314 participants provided no TLFB or UDS data. So that we could include these participants in the analysis, we assumed that each provided one day of data that indicated opioid use. The percentage of participants who were retained to endpoint but had periodic missing data for the primary outcome was 3.7%, so the censoring (exposure) variable, rather than multiple imputation, was used at all levels of retention to manage missing data.

### Interventions

In the SoC group, 155 (99.4%) of 156 study participants received their allocated drug. Among these, 123 (79.4%) of 155 participants were enrolled in ongoing treatment at the endpoint, or their treatment had been discontinued. During study treatment, 32 (20.6%) of these 155 participants presented for OUD treatment again and they were initiated on SoC maintenance (treatment was re-started on a total of 56 occasions). Data was available on SoC maintenance dose level for 137 [87.8%] of 156 participants who received Bup-SL (mean 12.0 mg/day [SD 6.1; range: 1–26 mg/day) and for 11 participants who received MET (mean 28.9 mg/day [SD 20.8; range 20–61 mg/day).

In the BUP-XR group, 150 (94.9%) of 158 participants received their allocated study drug. For the eight participants that did not receive study BUP-XR, four declined to receive, two did not complete the run-in, and two were arrested. During the study, we collected treatment data on seven of these eight participants—five received BUP-SL, one received MET, and one received BUP-SL and also MET at different times.

Among the 150 participants receiving BUP-XR, the mean number of injections received was 4.98 (SD 1.84). 110 (69.6%) of 158 participants received all six injections. There was an average of 24.3 days (SD 3.0; range: 20–41 days) between the two loading doses, and an overall average of 29.1 days (SD 2.11; range: 24.5–38.5 days) between the four maintenance doses. In error, one participant received their second loading dose on day 20 and this was recorded as a protocol deviation.

Among the 110 participants who received all six injections, the most common dosing profile was 2 × 300 mg then 4 × 100 mg (among 75 participants [68.2%] of these 110 participants). There were two distinct additional dosing patterns: 11 participants (10.0%) of 110 received 3 × 300 mg then 3 × 100 mg; and four participants (3.6%) of 110 received 6 × 300 mg. The remaining 20 participants had a mixed dosage pattern (Appendix I, Supplementary Fig. S1.1, page 6 for data visualisation of the dosing patterns). 13 participants increased their dose to 300 mg after dropping to 100 mg.

In three of five treatment clinics, eight (5.1%) of 158 participants in the BUP-XR group received supplemental BUP-SL medication (initial dose range: 2–8 mg). Six of these participants received supplemental dosing on one or more days in one inter-injection interval. Two received supplemental dosing on one or more days in two inter-injection intervals.

### Primary outcome

**Fig. 2:**
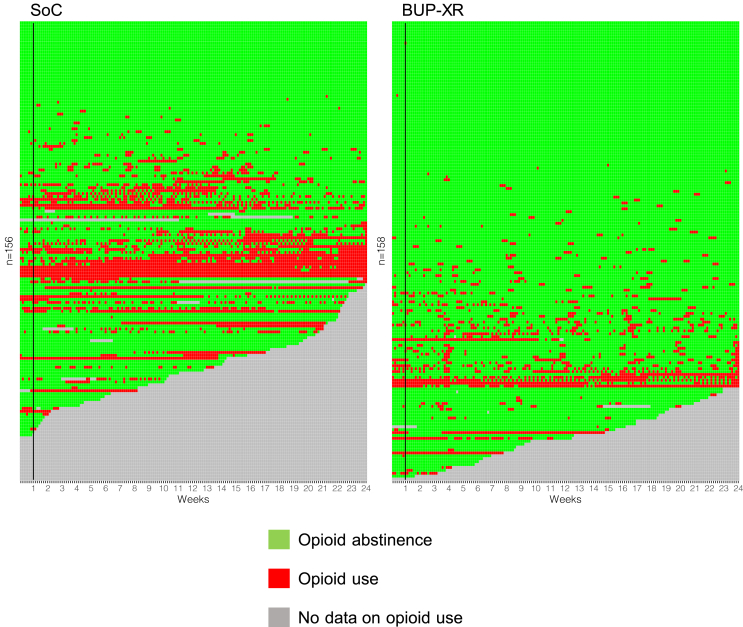
Longitudinal course of opioid use for each participant (full analysis set).

For the full analysis set (n = 314), there was an adjusted mean of 104.37 days (SE 9.89; range: 0–161 days) of opioid abstinence in the SoC group and 123.43 days (SE 4.76; range: 24–161 days) of opioid abstinence in the BUP-XR group (IRR 1.18; 95% 1.05–1.33; p-value 0.004). Fig. 2 displays a data visualisation of the unadjusted longitudinal course of day-by-day opioid use and abstinence by treatment group. All sensitivity checks on the primary endpoint analysis yielded statistically significant treatment estimates in favour of the BUP-XR group (IRR range 1.16–1.41; p-value 0.001–0.01) (Table 2). Protocol deviations stood at 18%; therefore the planned per-protocol analysis was undertaken. This excluded participants not receiving the randomised allocation and those not receiving treatment within 7 days (n = 28). The per-protocol analysis (n = 286) yielded a statistically significant BUP-XR treatment effect (IRR 1.21; 95% CI 1.04–1.41; p-value 0.02).

**Table 2:** Primary outcome and sensitivity analysis (full-analysis set).

Analysis	SoC (n = 156)	BUP-XR (n = 158)	Difference (SE)	Adjusted IRR (95% CI)	p-value
**Primary outcome measure**—mean days abstinent from opioids	104.37 (9.89)	123.43 (4.76)	19.05 (5.48)	1.18 (1.05–1.33)	0.004
**Primary outcome measure**—sensitivity analysis					
**Adjustment of analysis period**					
Including 1-week grace period^a^	111.86 (10.36)	130.44 (4.89)	18.58 (5.84)	1.16 (1.04–1.31)	0.008
TLFB data truncated by 14 days at endpoint^b^	96.72 (9.28)	114.37 (4.32)	17.65 (5.27)	1.18 (1.05–1.33)	0.006
Duration of treatment episode at study enrolment^c^	104.27 (9.71)	123.09 (4.68)	18.82 (6.03)	1.18 (1.05–1.32)	0.004
**Inclusion of baseline predictors of missing data**					
ADAPT ‘addiction severity’ score and QUID-SR score	102.47 (5.42)	119.92 (1.19)	17.45 (6.03)	1.17 (1.05–1.31)	0.007
**Missing TLFB data scenario**					
Best case—all missing data indicate opioid abstinence	131.19 (11.00)	152.34 (3.56)	21.15 (7.85)	1.16 (1.03–1.31)	0.018
Worst case—all missing data indicate opioid use	94.37 (7.59)	133.41 (7.28)	39.04 (10.40)	1.41 (1.17–1.71)	0.001
**Missing UDS data scenario**					
Best case—all missing data indicate opioid abstinence	122.04 (12.61)	150.06 (4.55)	28.02 (9.15)	1.23 (1.05–1.44)	0.009
Worst case—all missing data indicate opioid use	121.98 (12.68)	149.96 (4.65)	27.98 (9.16)	1.23 (1.05–1.44)	0.010

All analysis is mixed-effects, negative binomial regression with baseline drug injecting status (fixed effect) and treatment clinic (random intercept).

Difference is calculated as BUP-XR minus SoC.

ADAPT, Addiction Dimensions for Assessment and Personalised Treatment; BUP-XR, extended-release buprenorphine; IRR, incidence rate ratio; QIDS-SR, Quick Inventory of Depressive Symptomatology–Self-Report; SE, standard error; SoC, the standard of care; TLFB, Timeline Follow-back; UDS, urine drug screen.

All means are adjusted.

a Analysis period is day 0–168.

b Analysis period is day 8–154.

c Days in treatment at study enrolment.

In the sub-groups analysis (Table 3), participants with more severe OUD-related problems on the CGI-S at baseline achieved less days of opioid abstinence overall (IRR 0.68; 95% CI 0.52–0.88; p-value 0.004). Inclusion of this sub-group in the analysis model reduced the BUP-XR treatment effect (IRR 1.06; 95% CI 1.01–1.12; p-value 0.029). A statistically significant treatment group by CGI–S interaction (IRR 1.32; 95% CI 1.25–1.39; p-value 0.001; mean scores displayed for interpretation in Table 3), indicated that the both CGI-S groupings had more abstinent days in the BUP-XR treatment group, and that the treatment effect observed for participants rated more severe at baseline was relatively greater in the BUP-XR group.

**Table 3:** Sub-group analyses on primary outcome (full-analysis set).

Analysis—baseline sub-group	SoC (n = 156)	BUP-XR (n = 158)	Adjusted IRR (95% CI)	p-value
**CGI-S**—extremely mild–mild vs. moderate–extremely severe				
Treatment group	102.84 (6.19)	121.48 (4.91)	1.06 (1.01–1.12)	0.029
CGI-S			0.68 (0.52–0.88)	0.004
Interaction			1.32 (1.25–1.39)	0.001
CGI-S (extremely mild–mild)	119.97 (2.66)	127.17 (1.73)		
CGI-S (moderate–extremely)	81.53 (11.82)	114.23 (11.88)		
**Cocaine use**—use in past 28 days				
Treatment group	104.59 (7.59)	122.74 (5.07)	1.05 (0.99–1.12)	0.092
Cocaine use	118.10 (3.56)	124.59 (3.48)	0.78 (0.55–1.11)	0.172
Interaction			1.24 (0.94–1.63)	0.124
**Benzodiazepines**—use in past 28 days				
Treatment group	104.35 (9.93)	123.42 (4.79)	1.19 (1.04–1.35)	0.010
Benzodiazepine use	104.10 (10.82)	123.41 (5.22)	1.01 (0.93–1.10)	0.827
Interaction			0.99 (0.91–1.07)	0.821
**Time in treatment before study enrolment**—less than 28 days vs. 28 days or more				
Treatment group	104.09 (8.44)	122.11 (5.59)	1.13 (1.08–1.18)	0.001
Time (longer)	109.91 (4.56)	124.07 (3.09)	0.80 (0.54–1.19)	0.271
Interaction			1.18 (0.94–1.46)	0.144
**COVID-19 restrictions**—recruitment before vs. after^a^				
Treatment group	104.53 (10.02)	123.70 (4.67)	1.19 (1.06–1.34)	0.004
Recruitment before restrictions	108.35 (9.21)	126.62 (3.36)	1.05 (0.96–1.14)	0.271
Interaction			0.98 (0.91–1.07)	0.684

Analysis period is day 0–168; All means adjusted.

All analysis is mixed-effects, negative binomial regression with baseline drug injecting status (fixed effect), treatment clinic (random intercept), and treatment group x sub-group interaction.

BUP-XR, extended-release buprenorphine; CGI-I, Clinical Global Impression–Severity; IRR, incidence rate ratio; SoC, standard-of-care.

a Recruitment paused in first study clinic on 20 March 2020 (n = 76); recruitment resumed in all study clinics on 12 June 2020 (n = 238).

### Secondary outcomes

The adjusted total mean days of enrolment in study treatment was 128.5 days (SE 4.82) in the SoC group and 144.6 days (SE 2.54) in the BUP-XR group (IRR 1.12; 95% CI 1.01–1.25; p-value 0.029). With censoring, the average number of days to discontinuation was 138.2 days (SD 47.7) in the SoC group and 154.0 days (SD 33.6) in the BUP-XR group (hazard ratio 0.46; 95% CI 0.33–0.66; p-value <0.001). The proportional hazards assumption held as evidenced by a non-significant treatment-by-time interaction (p-value 0.826).

In mixed-effects logistic regression, with a fixed effect for treatment group and the drug injecting stratification variable, a random intercept for treatment clinic, and using multiple imputation for missing data, the adjusted probability of OUD remission at the 12-week follow-up was 0.50 (SE 0.004) for the 156 participants in the SoC group and 0.73 (SE 0.003) for the 158 participants in the BUP-XR group (adjusted odds ratio [OR] 2.69; 95% CI 1.50–4.89; p-value 0.001). The adjusted probability of early OUD remission for all 24 weeks of follow-up was 0.62 (SE 0.001) in the SoC group and 0.75 (SE 0.001) in the BUP-XR group (OR 1.90; 95% CI 1.02–3.52; p-value 0.042).

Among participants reporting use of cocaine at baseline (148 participants in the SoC group and 147 participants in the BUP-XR group), there was no treatment effect for CUD early remission at either follow-up (0.48 [SE 0.02] in the SoC group and 0.52 [SE 0.02] in the BUP-XR group at 12-weeks [OR 1.09; 95% CI 0.59–2.00; p-value 0.785]; and 0.53 [SE 0.02] in the SoC group and 0.43 [SE 0.02] in the BUP-XR group at 24-week follow-up [OR 0.59; 95% CI 0.31–1.11; p-value 0.103]).

**Fig. 3:**
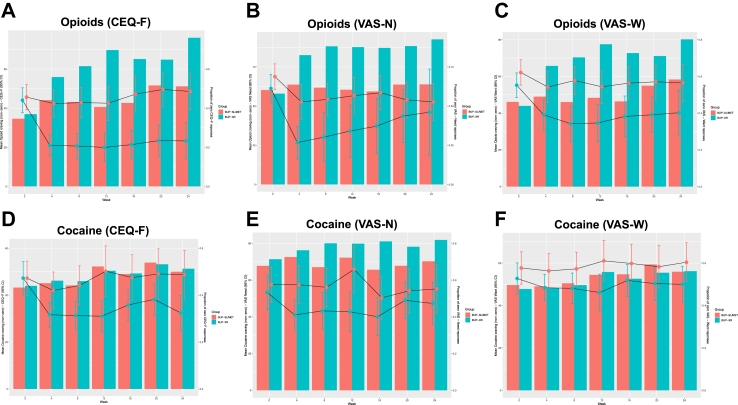
Craving for opioids and cocaine by group and week (full-analysis set).

The other secondary outcomes are shown in Table 4. From an approximately equal baseline, Fig. 3 panel A–F displays the longitudinal response on the CEQ-F, VAS-N and VAS-W, separating the proportion of each treatment group by follow-up week with a zero response, and the mean response among participants who did experience craving (i.e., a non-zero response on the scale). This unexpected pattern caused pronounced skew, so we used a zero-inflated Poisson approach to enable two-part modelling of the observed distributions at the endpoint. Both parts of the model were fitted with the drug injecting stratification variable, the outcome measure’s baseline score, and treatment group (all fixed effects). For opioids, there was a treatment effect for BUP-XR on the CEQ-F indicating a statistically significant likelihood of a zero score and a lower mean score relative to the SoC group (Fig. 3 panel A–C; Table 4). This effect was also estimated for the likelihood of a zero score on the VAS-N and VAS-W, but it was not found for a difference in the non-zero mean score.

**Table 4:** Analysis of secondary outcomes (full-analysis set).

Outcome	SoC (n = 156)	BUP-XR (n = 158)	Adjusted estimate (95% CI)	p-value
**Clinical assessments**				
**Craving for opioids**^c^				
Zero score on CEQ-F—probability	0.54 (0.04)	0.92 (0.03)	OR 3.22 (1.65–6.36)	0.001
Non-zero score on CEQ-F—mean	20.87 (2.66)	5.80 (1.52)	IRR 0.52 (0.34–0.81)	0.004
Zero score on VAS-N—probability	0.67 (0.04)	0.92 (0.02)	OR 6.17 (2.61–14.45)	0.001
Non-zero score on VAS-N—mean	17.24 (2.27)	3.75 (1.21)	IRR 0.83 (0.59–1.15)	0.248
Zero score on VAS-W—probability	0.60 (0.05)	0.79 (0.04)	OR 3.00 (1.20–7.39)	0.018
Non-zero score on VAS-W—mean	21.81 (3.43)	8.08 (2.24)	IRR 0.70 (0.49–1.00)	0.052
**Craving for cocaine**^c^				
Zero score on CEQ-F—probability	0.52 (0.05)	0.51 (0.04)	OR 0.94 (0.41–2.16)	0.885
Non-zero score on CEQ-F—mean	21.90 (3.29)	15.84 (2.53)	IRR 0.71 (0.46–1.10)	0.124
Zero score on VAS-N—probability	0.74 (0.05)	0.81 (0.04)	OR 1.59 (0.53–4.81)	0.400
Non-zero score on VAS-N—mean	13.26 (2.37)	9.57 (2.30)	IRR 0.97 (0.77–1.23)	0.814
Zero score on VAS-W—probability	0.58 (0.05)	0.55 (0.04)	OR 0.85 (0.38–1.97)	0.707
Non-zero score on VAS-W—mean	24.11 (2.83)	21.79 (2.28)	IRR 0.85 (0.77–1.02)	0.077
**Opioid use**				
Maximum days continuously abstinent—mean	77.44 (12.34)	95.08 (8.50)	IRR 1.23 (1.06–1.42)	0.005
**Cocaine use**				
Days abstinent—mean	102.89 (11.90)	112.16 (5.91)	IRR 1.09 (0.95–1.25)	0.230
Maximum days continuously abstinent—mean	70.45 (12.33)	71.34 (9.21)	IRR 1.01 (0.86–1.19)	0.877
**Benzodiazepine use**				
Days abstinent—mean	115.06 (10.39)	121.16 (5.64)	IRR 1.05 (0.95–1.16)	0.312
Maximum days continuously abstinent—mean	92.65 (12.04)	104.04 (7.85)	IRR 1.12 (0.10–0.94)	0.198
**Alcohol use**				
Drinking days—mean^b^	1.36 (0.25)	1.25 (0.24)	IRR 0.88 (0.51–1.52)	0.640
Standard units of alcohol—log transformed mean^a^	1.79 (0.14)	1.89 (0.12)	Difference 0.09 (0.22–0.40)	0.545
Maximum units on drinking day—log transformed mean^a^	2.01 (0.21)	2.11 (0.15)	Difference 0.11 (−0.24 to 0.45)	0.545

Outcome	SoC (n = 156)	BUP-XR (n = 158)	Adjusted estimate (95% CI)	p-value
**Clinical assessments—continued**				
DERS-SF—mean^f^	45.13 (1.68)	42.52 (0.96)	Difference −2.61 (−6.12 to 0.91)	0.145
QUIDS-SR—mean^f^	7.49 (0.56)	6.75 (0.50)	Difference −0.74 (−1.86 to 0.38)	0.194
WSAS—mean^g^	−4.45 (1.38)	−8.25 (1.02)	Difference −3.80 (−6.76 to −0.84)	0.012
**Clinician-reported**				
ADAPT				
OUD severity—mean^g^	−0.49 (0.36)	−0.77 (0.30)	Difference −0.28 (−0.70 to 0.136)	0.185
Concurrent problem complexity—mean^g^	−0.64 (0.47)	−1.09 (0.37)	Difference −0.45 (−1.34 to 0.44)	0.322
Recovery strengths—mean^g^	−0.27 (0.32)	0.29 (0.27)	Difference 0.55 (0.07–1.05)	0.025
CGI-I—proportion not changed or worsened^d^	0.46 (0.01)	0.10 (0.001)	OR 0.12 (0.05–0.27)	0.001
**Patient-reported**				
PRO-I—proportion not changed or worsened^d^	0.32 (0.01)	0.10 (0.002)	OR 0.21 (0.09–0.47)	0.001
SURE—mean^e^	51.30 (1.40)	56.03 (1.13)	Difference 6.28 (3.58–8.98)	0.001

Data in parentheses are standard error, percentage or 95% confidence interval; all means are adjusted.

ADAPT, Addiction Dimensions for Assessment and Personalised Treatment; ALC-QFM, alcohol consumption scale: frequency, quantity, and maximum quantity of alcohol consumed on any one day; BUP-XR, extended-release injectable buprenorphine; CEQ-F, Craving Experiences Questionnaire-frequency version; CGI-I, Clinical Global rated evaluation of improvement (minimally improved–very much improved vs. no change–very much worse); DERS-SF, Difficulties in Emotion Regulation Scale-Short Form; HR, hazard ratio; IRR, incidence rate ratio; VAS-N/W, visual analogue scale of perceived need and want for drug; OR, odds ratio; PRO-I, Patient Reporting Outcome—Improvement (minimally improved–very much improved vs. no change–very much worse); less than moderate versus moderate and above); SoC, standard-of-care; QIDS-SR, Quick Inventory of Depressive Symptomatology-Self-Report; WSAS, Work and Social Adjustment Scale.

a Mixed-effects negative binomial regression, with stratification factors and baseline score (fixed effects) and clinic (random intercept) with log transformation of average and maximum number of units.

b Mixed-effects negative binomial regression, with stratification factor and baseline score (fixed effects) and clinic (random intercept).

c Mixed-effects zero-inflated Poisson regression, with stratification factor and baseline score and treatment clinic (fixed effects).

d Mixed-effects logistic regression, with stratification factor and baseline score (fixed effect) and treatment clinic (random intercept).

e Mixed-effects tobit regression, with stratification factor (fixed effect) and treatment clinic (random intercept).

f Mixed effects general linear model, with stratification factor, baseline score (fixed effects) and treatment clinic (random intercept).

g Mixed-effects general linear model on change from baseline, with stratification factor (fixed effect) and treatment clinic (random intercept).

There was no statistically significant treatment group effect for cocaine craving (Fig. 3 panel D–F; Table 4). There was no statistically significant evidence for a treatment effect on the use of cocaine or benzodiazepine use. As a post-hoc check, there was no evidence for any compensatory cocaine use during follow-up, but there was modest evidence of an increase in benzodiazepine use in the BUP-XR group (Appendix I, Supplementary Table S1.2, page 3).

Due to sparseness of response to some categories in the CGI-I and PRO-I (observed data in Appendix I, Supplementary Table S1.3, page 4), we classified participants into two groups (‘minimally improved–very much improved’ versus ‘no change–very much worse’). Normality assumptions were not met for the ADAPT, ALC-QFM, SURE and WSAS endpoint scores, and the SURE also demonstrated a ceiling effect. The following deviations to the SAP were made: the ADAPT and WSAS were analysed by change score from baseline; the ALQ-QFM was analysed by negative binomial regression, with a log transformation applied to the average and maximum number of standard units; and a mixed-effects tobit regression was used for the analysis of the SURE.

There was evidence of a BUP-XR treatment effect for the recovery strengths sub-scale on the ADAPT, the SURE and WSAS, and greater odds of improvement on the CGI-I and PRO-I. There was no discernible treatment effect for the endpoint alcohol use measures, or for DERS-SF and QUID-SR outcomes.

### Safety

The incidence of any adverse event was higher in the BUP-XR group than the SoC group (128 [81.0%] of 158 participants versus 67 [42.9%] of 156 participants, respectively [Table 5; all events in Appendix I, Supplementary Table S1.4, page 5]). The most common adverse event in the BUP-XR group was rated in a mild–moderate range and rapidly-resolving pain from drug administration (121 [26.9%] of 450 adverse events). In the SoC group, the most common adverse events were infections and infestations (38 [28.6%] of 133 adverse events).

**Table 5:** Safety events (full analysis set).

Safety event	SoC (n = 156)	BUP-XR (n = 158)
**Treatment-emergent adverse events**		
Number of participants reporting	67 (42.9)	128 (81.0)
Number of adverse events	133	450
Type of adverse event—number^a^		
Drug administration (pain and pruritis)	4 (3.0)	121 (26.9)
Gastrointestinal disorders	13 (9.8)	61 (13.6)
Nervous system disorders	2 (1.5)	42 (9.3)
Psychiatric disorders	20 (15.0)	32 (7.1)
Infections and infestations	38 (28.6)	34 (7.6)
Musculoskeletal and connective tissue disorders	9 (6.8)	35 (7.8)
Injury, poisoning, and procedural complications	13 (9.7)	22 (4.9)
Skin and subcutaneous tissue disorders	5 (3.8)	24 (5.3)
Unintentional drug poisoning adverse events		
Total number of participants reporting	3 (2.3)	3 (0.7)
Drug class—benzodiazepines	0 (−)	1 (0.2)
Drug class—not known	3 (2.3)	2 (0.4)
**Treatment-emergent serious adverse events**		
Total number of participants reporting	18 (11.5)	11 (7.0)
Total number of serious adverse events	26	14
Type of serious adverse event—number		
Infections and infestations	0 (−)	1 (7.1)
Neoplasms benign, malignant and unspecified	0 (−)	1 (7.1)
Immune system disorders	1 (3.9)	0 (−)
Psychiatric disorders	2 (7.7)	2 (14.3)
Nervous system disorders	6 (23.1)	1 (7.1)^b^
Vascular disorders	1 (3.9)	1 (7.1)
Gastrointestinal disorders	2 (7.7)	0 (−)
Hepatobiliary disorders	0 (−)	2 (14.3)
Skin and subcutaneous tissue disorders	1 (3.9)	0 (−)
Musculoskeletal and connective tissue	1 (3.9)	0 (−)
Renal and urinary disorders	0 (−)	1 (7.1)
Pregnancy, puerperium and perinatal	1 (3.9)	1 (7.1)
Injury, poisoning and procedural complication	7 (26.9)	1 (7.1)
Surgical and medical procedures	0 (−)	1 (7.1)
Social circumstances	1 (3.9)	0 (−)
Inconclusive by Coroner	1 (3.9)^b^	0 (−)
Unintentional drug poisoning serious adverse events^c^		
Total number of participants reporting	2 (7.7)	2 (14.3)
Drug class—opioids	1 (3.9)	1 (7.1)
Drug class—benzodiazepines	1 (3.9)	0 (−)
Drug class/type—benzodiazepines and cocaine	0 (−)	1 (7.1)

Data are number (%); Appendix I, Supplementary Table S1.4, page 5) shows all adverse events.

BUP-XR, extended-release buprenorphine; SoC, standard-of-care.

a Reported in at least 5% of participants.

b Participant died.

c All discharged after hospital treatment.

There were 11 serious adverse events (7.0%) in the 158 participants in the BUP-XR group, and 18 serious adverse events (11.5%) in the 156 participants in the SoC group. No adverse event was judged to be related to study treatment. Two participants died: one in the SoC group, on which the coroner reported an inconclusive cause of death; one in the BUP-XR group due to nervous system disorder (brain abscess). Four participants (two in each treatment group) were treated at the Accident and Emergency Department following unintentional drug poisoning and discharged.

### Cost-effectiveness

The economic evaluation was done on the full analysis set (n = 314). The following deviations to the HEAP were made: criminal justice costs were aggregated by follow-up point prior before imputation due to problems with model convergence, and treatment clinic and the drug injection stratification variable were fit with random intercepts.

There was a higher proportion of missing data in the SoC group (Appendix II, Supplementary Table S2.4, page 20). The observed frequency of resource use at each time point over the 24-weeks of follow-up—including short and longer keyworker contacts recorded by KCF are shown in Appendix II (Supplementary Table S2.5, page 22). Disaggregated and total unadjusted mean costs and distribution of participants’ responses to each EQ-5D attribute and utility scores during follow-up are shown in Appendix II (Supplementary Table S2.6, page 24; Supplementary Table S2.7, page 26, and Supplementary Fig. S2.2, page 25).

**Fig. 4:**
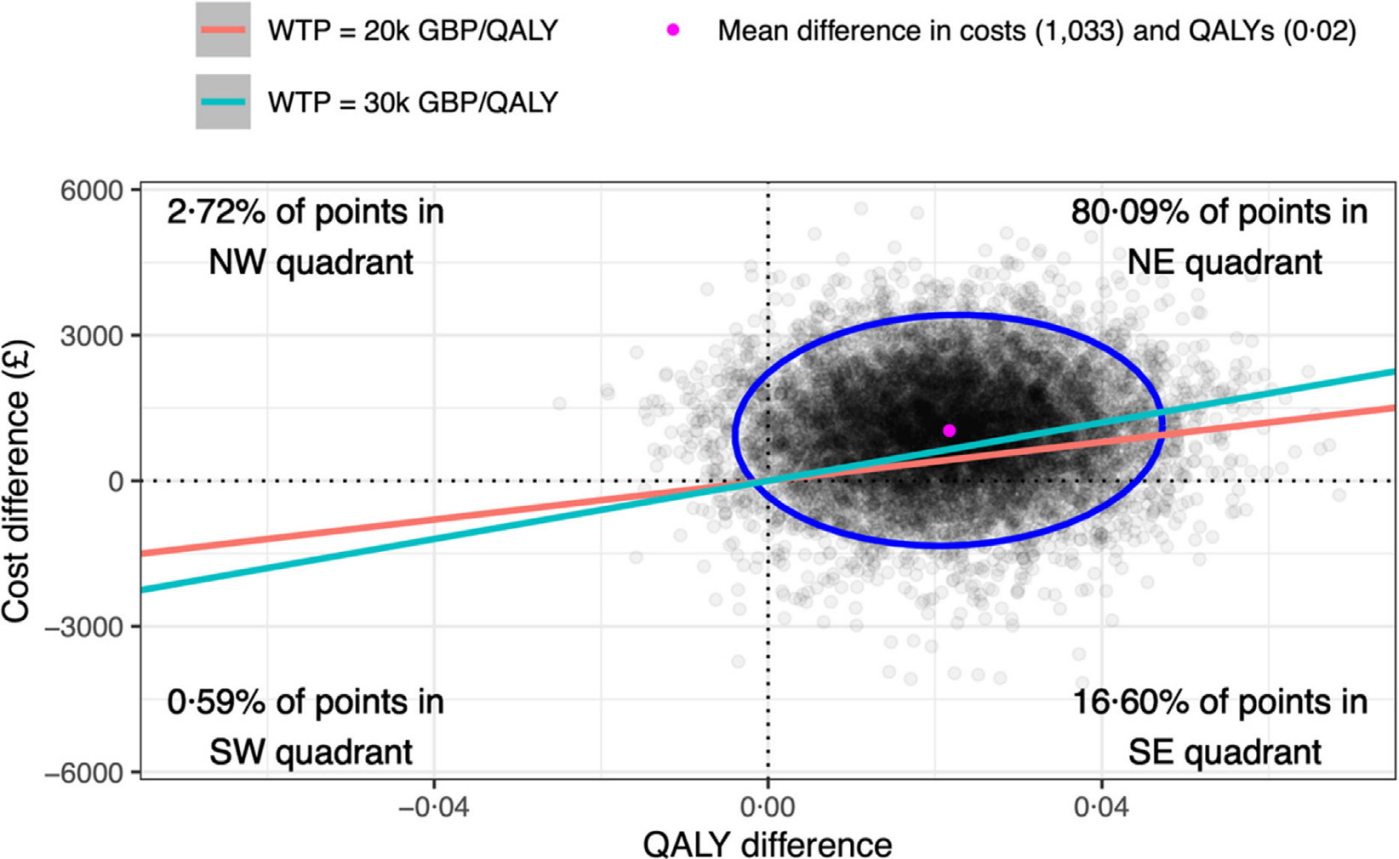
Cost-effectiveness plane showing the bootstrapped mean differences and 95% confidence ellipse in imputed total adjusted costs and QALYs of BUP-XR compared to the SoC.

BUP-XR was more costly than the SoC, with incremental costs of £1033 (95% CR −1189 to 3225), and more effective, with incremental QALYs of 0.02 (95% CR 0.00–0.05). For the base case, the resulting ICER was £47,540 per QALY gained (Table 6). 80.09% of the bootstrapped results fell in the north-east quadrant of the cost-effectiveness plane and the probability of being cost-effective at a WTP of £30,000 per QALY gained was 0.37 (Fig. 4; Appendix II, Supplementary Figs. S2.3 and S2.4, page 27).

**Table 6:** Summary of cost-utility analysis for base case, and by scenario and sub-group.

Analysis	Cost difference	QALY difference	INMB @ £20 k/QALY	INMB @ £30 k/QALY	ICER
Base case^a^	1033 (−1189 to 3225)	0.02 (0.00–0.05)	−598 (−2832 to 1681)	−381 (−2663 to 1976)	47,540
**Scenario**					
EQ-VAS^a^	1114 (−1090 to 3286)	0.03 (0.01–0.05)	−561 (−2716 to 1654)	−285 (−2462 to 1949)	40,310
Assume only MET used^a^	859 (−1761 to 3033)	0.02 (0.00–0.05)	−420 (−2627 to 2223)	−201 (−2428 to 2481)	39,167
Lower BUP-XR cost (£239.70)^a^	896 (−1305 to 2981)	0.02 (0.00–0.05)	−462 (−2608 to 1738)	−246 (−2475 to 1965)	41,324
BUP-SL/MET daily supervised^a^	839 (−1375 to 2959)	0.02 (0.00–0.05)	−406 (−2572 to 1805)	−190 (−2417 to 2098)	38,781
BUP-SL/MET fortnightly unsupervised^a^	1155 (−1018 to 3332)	0.02 (0.00–0.05)	−720 (−2960 to 1539)	−503 (−2794 to 1795)	53,111
Best case^a^, ^b^	573 (−1703 to 2595)	0.02 (0.00–0.05)	−141 (−2234 to 2094)	75 (−2097 to 2344)	26,546
Worst case^a^, ^c^	1436 (−792 to 3660)	0.02 (0.00–0.05)	−1003 (−3212 to 1253)	−787 (−3088 to 1511)	66,383
NHS + PSS + CJ + A/Base case	1001 (−1209 to 3186)	0.02 (0.00–0.04)	−576 (−2793 to 1698)	−363 (−2596 to 1977)	47,047
NHS + PSS + CJ/Base case	1300 (−659 to 3377)	0.02 (0.00–0.04)	−885 (−3043 to 1089)	−678 (−2892 to 1330)	62,717
NHS + PSS/Base case	944 (−136 to 2264)	0.02 (0.00–0.04)	−522 (−1895 to 607)	−312 (−1701 to 906)	44,785
NHS + PSS/Lower BUP-XR cost (£239.70)	786 (−275 to 2018)	0.02 (0.00–0.04)	−365 (−1636 to 761)	−154 (−1503 to 1053)	37,323
NHS/Base case	962 (−93 to 2286)	0.02 (0.00–0.04)	−534 (−1879 to 589)	−320 (−1764 to 868)	44,955
**Sub-group**					
Use of cocaine^a^	3062 (−3553 to 10,435)	0.02 (−0.03 to 0.08)	−2623 (−10,023 to 4044)	−2404 (−9821 to 4407)	139,469
Use of benzodiazepines^a^	3500 (−5588 to 13,181)	0.03 (−0.03 to 0.11)	−2854 (−12,431 to 6483)	−2531 (−12,216 to 7005)	108,356
CGI-S rating severe^a^	−3147 (−11,435 to 4149)	0.05 (−0.01 to 0.11)	4136 (−3262 to 12,391)	4630 (−2876 to 12,982)	Dominant
Treatment episode > 28 days^a^	−102 (−4368 to 3370)	0.02 (−0.01 to 0.05)	576 (−2926 to 4850)	812 (−2779 to 5089)	Dominant

Data are mean (95% central range); Estimates from bootstrapped and imputed data, adjusted for baseline costs and utilities, site, and injecting status.

BUP-SL, sublingual tablet buprenorphine; INMB, Incremental Net Monetary Benefits; MET, oral liquid methadone.

a Societal perspective; NHS + PSS + CJ + A is NHS is Personal Social Services, Criminal Justice, and Accommodation perspective.

b BUP-SL/MET at February 2019 prices (inflated to 2021 prices using HCHS index) with assumption of six supervised medication pick-ups per week.

c BUP-SL/MET at July 2018 prices (inflated to 2021 prices using HCHS index) and assuming one unsupervised medication pick-up per fortnight.

Results of the sensitivity analyses indicated that the cost of trial medication (followed by costs of concomitant medication, accommodation, and contacts with the criminal justice system) had the greatest impact on INMB. There was no single cost input that, when varied by ±10%, would have changed the cost-effectiveness decision at the £30,000 per QALY gained threshold (Appendix II, Supplementary Fig. S2.5, page 28).

Among the 12 scenario analyses, ICERs were all higher than £30,000 per QALY except for a ‘best case’ scenario from the societal perspective: with February 2019 prices for BUP-SL/MET inflated to 2021 prices using the HCHS index, and an assumption that six doses were administered under supervision each week, the ICER was £26,546 by per QALY gained (Table 6; Supplementary Fig. S2.6, page 31).

The results of the sub-group analyses suggest that BUP-XR would not be cost-effective for patients who were using benzodiazepines or cocaine (ICERs > £100 k per QALY gained for both subgroups), but that BUP-XR dominated SoC in participants whose baseline CGI-S rating was ‘moderate-extremely severe’, or whose current treatment episode was greater than 28 days (Table 6; Appendix II, Supplementary Table S2.9, page 32).

## Discussion

Meeting the primary endpoint, this study has secured first evidence for BUP-XR superiority compared with the SoC. Conducted in standard clinical conditions in the NHS, with no media recruitment of participants, the results generalise to the populations seeking treatment and those enrolled in the SoC in the UK NHS. During the 24 weeks of study treatment, on average participants allocated to BUP-XR achieved an additional 19 days of opioid abstinence. They also achieved longer continuous opioid abstinence (95.1 versus 77.4 days), had longer time enrolled in treatment (144.6 versus 128.5 days), and had longer time to (any) discontinuation of treatment (154.0 versus 138.2 days).

The BUP-XR treatment effect for the primary outcome was maintained in all sensitivity models (IRR effect range 1.16–1.41; p-value 0.001–0.01). The treatment effect was not reduced in all sub-group analyses, and participants in the BUP-XR group who were clinician rated with moderate–severe OUD-related problems at the outset achieved more opioid abstinence (114.2 versus 81.5 days).

Among the secondary outcomes, the BUP-XR group were more likely to be in early OUD remission at 24 weeks, experience a greater suppression and reduction in opioid craving, and achieve higher patient-reported and clinician-reported evaluations across all clinical scales bar the two addressing emotion dysregulation and depression symptomatology. These outcomes will be further investigated in our mixed-methods PSI study.

BUP-XR was acceptable to the majority, with 110 [69.6%] of 158 participants attending as invited for all six injections according to the dosing regimen. Some participants did request supplementary BUP-SL medication between maintenance injections (8 [5.1%] of 158 participants). Our clinical experience while running the study was that participants appreciated the relatively wide inter-injection windows so they could schedule their time to visit the clinic. Patient experiences will now be investigated in our qualitative study.

The safety profile of BUP-XR was comparable to the SoC. There were more adverse events recorded for BUP-XR group but reflected transient mild–moderate pain during drug administration. There was no discernible treatment group difference in the incidence and type of serious adverse events. All serious adverse event were judged to be unrelated to study treatment—with the exception that one participant in the BUP-XR group’s diagnosis of schizophrenia (was judged unlikely to be related to study treatment). There was one death in each treatment group that was judged unrelated to study treatment. Two participants in each group were treated at the Accident and Emergency department for unintentional drug poisoning and then discharged.

From a societal cost perspective, the base-case economic analysis showed that BUP-XR had an ICER of £47,540 per QALY gained, and regardless of the perspective taken, it was not cost effective at the WTP thresholds per QALY gained that are normally considered by NICE in technology appraisals. This finding was robust in all analyses, except in the scenario where both oral medication acquisition and dispensing costs were high for all SoC participants (ICER for BUP-XR was £26,546 by per QALY gained). In sub-group analyses, BUP-XR was cost-effective (dominated) among participants with severe problems at study baseline, or those whose duration of treatment was greater than 28 days.

Baseline EQ-5D utilities for EXPO participants (0.74 in both treatment groups) were also higher than the 0.43–0.68 range reported in two previous OUD treatment trials,^29^^,^^42^ so there may have been less room for improvement in the EXPO sample than in the wider OUD population. The hypothesis is congruent with the severity sub-group findings (i.e., a more severe rating of OUD-related problems at baseline was linked to more opioid abstinence).

Previous OUD intervention studies have shown economic benefits to relate to reduced crime and victim of crime costs.^29^ This was observed in EXPO—but the reduced overall criminal justice costs in favour of BUP-XR during follow-up was not statistically significant. Somewhat counter-intuitively, the scenario analyses suggest that including criminal justice costs reduce the probability of BUP-XR being cost-effective. This was due to an imbalance in costs at baseline, where mean total observed costs for criminal justice were ∼£310 lower for the BUP-XR group than the SoC group.

Results of this and other economic analyses of BUP-XR (Sublocade® and Buvidal®) appear to be sensitive to medicine acquisition and administration costs, both of BUP-XR and the comparator, typically BUP or BUP-NLX. In Scotland, a Markov-model based analysis of Buvidal® and BUP-NLX with a one-year time horizon, with equal outcomes assumed, and with costs limited to medicine acquisition, administration/pharmacy, and other health resource use, Buvidal® was considered cost-saving (−£140); however, changing the comparator from BUP-NLX to mono BUP reversed this, with an incremental cost of £213.^51^ In Wales, the All-Wales Medicine Strategy Group assessment for Buvidal® suggests that it dominates BUP-NLX^52^ based on the assumption that Buvidal® would have lower administration costs.

What might the underlying mechanism be for BUP-XR’s comparative clinical superiority? It is likely that several processes are at work and further studies are needed—but the levels of BUP exposure driving BUP μ-OR occupancy may turn out to be key. A clinically responding patient should not experience any opioid withdrawal symptoms due to BUP agonist properties; in the past, onset of these symptoms may well have initiated a cognitive-affective cycle of distress and motivated drug-seeking and consumption. Therapeutic control of withdrawal symptoms has been found to be achieved with BUP plasma concentration levels ≥1 ng/mL (corresponding to ≥ 50% μ-OR occupancy).^53^ A second likely mechanism of effect is through the blockade of the subjective (euphoric) effects of opioids, so that any lapsed drug use is not directly reinforcing. Opioid blockade is best achieved by BUP plasma levels ≥ 2–3 ng/mL occupying ≥70% of μ-OR receptors.^54^ A study that had pooled phase II and phase III studies with a total of 570 participants treated for up to one year estimated that the BUP-XR 300/100 mg dosing regimen achieves and sustains BUP plasma levels ≥ 2–3 ng/mL over the inter-dosing interval, enabling both control of withdrawal symptoms and opioid blockade.^55^ This dual control of withdrawal symptoms (for all patients) and blockade of euphoria (for those lapsing reducing drug liking) translates to greater potential for cognitive control over craving and drug seeking, as supported by other clinical data.^56^

The primary endpoint and secondary opioid use outcomes were highlighted strikingly in the data visualisation (Fig. 2). In the BUP-XR group, the heatmap also indicated a much larger proportion of retained participants with a sporadic (or perhaps repeating) pattern of occasional opioid use; and a larger group of non-responding participants in the SoC with an almost–daily pattern of opioid use who were nonetheless retained to the study endpoint. Nevertheless, there was continued opioid use among some participants. We will now conduct causal-effect modelling of these longitudinal data which can leverage heatmap data for cocaine and benzodiazepine use to investigate patient and treatment factors that are linked to response and non-response to inform measurement-based care for OUD treatment.^57^

We had expected that these features of BUP-XR would translate to a progressively improved response on the CEQ-F and the VAS-N and VAS-W measures. This was borne out in the analysis, but surprisingly, there was clear evidence of a bimodal distribution on the CEQ-F separating responses into a dominant and increasing trend over time for complete suppression of craving (treatment effect at endpoint: OR 3.22; 95% CI 1.65–6.36; p-value 0.001) and a lower mean score relative to SoC among the relatively small group of participants that did experience craving (Fig. 3 panel A).

A two-process craving response was also seen in the VAS-N and VAS-W (Fig. 3 panel B and C), with a shift to a zero report of any ‘need’ or ‘want’ for opioids that was sustained across follow-up (treatment effect at endpoint: OR 6.17; 95% CI 2.61–14.45; p-value 0.001 and OR 3.00; 95% 1.20–7.39; p-value 0.018, respectively). However, in the relatively small group of participants that did experience of ‘need’ or ‘want’ their mean scores increased over time with no statistically significant treatment effect at the endpoint (IRR 0.83; 95% CI 0.59–1.15; p-value 0.248 and IRR 0.70; 95% CI 0.49–1.00; p-value 0.052, respectively). The relatively small group size for the ratings of drug ‘need’ and ‘want’ caution against over-interpretation of differences between multiple and single construct approaches to assessing craving which will be investigated further in a subsequent report. In the clinic, it may prove necessary to offer higher dose BUP-XR during maintenance in an effort to reduce bothersome craving and for those continuing to use non-medical opioids.

While there is no plausible direct effect mechanism for BUP-XR on cocaine (and other stimulant) use, we had expected that the opioid blockade effect of BUP-XR might make cocaine use aversive. The exploratory analyses of cocaine craving, cocaine use, and CUD remission did not show this. The search for an effective pharmacotherapy for CUD that might appeal to patients with dual OU-CUD has proved to be a very long road with many repurposed drugs or drug combinations evaluated but proving ineffective. Currently, prescription stimulants appear to hold promise,^58^ and research on novel compounds is gaining momentum.^59^ In the UK context, with the high prevalence of dual OUD-CUD the need to develop a pharmacotherapy for CUD gives a strong impetus for future studies of combined BUP-XR and investigational compounds. In EXPO, there was also no impact of study treatment on non-medical benzodiazepines and there is a priority need for effective pharmacotherapies, especially in high-prevalence countries.

Study findings have several limitations. Firstly, although it is very commonly used in treatment studies, a 24-week endpoint is a relatively short-term horizon to evaluate effectiveness or cost-effectiveness for a persistent disorder such as OUD. Observational studies in the USA and Australia have reported encouraging outcomes across opioid abstinence, and various indices of personal, social and occupational functioning between one-year^60^ and one-two years.^61^ Further studies are needed to determine if longer-term BUP-XR is effective at reducing opioid use and helping patients achieve and improvements in their personal and social functioning in the context of time-varying environmental and socio-economic factors.

Second, although the primary outcome measure was fit for the purpose of yielding a continuous capture of abstinence, it has not been widely used before. That said, the field lacks a gold-standard primary outcome measure to capture treatment response; there are dozens of variations in the way studies have operationalised treatment efficacy. Aside from more straightforward time-to-event time survival outcomes that are used to assess relapse, two broad approaches are common: a binary classification of treatment ‘responder’, and a count-based quantification of opioid abstinence as used here. There can be problems with the former given the risk of classifying ‘non-responders’ who achieve good but not-good-enough outcome, so we believe a count-based outcome is generally preferable. The inclusion of PRO measures—as we have done here—has also been recommended as a component of a core outcome set for OUD treatment research will make OUD treatment research more comparable.^62^

Third, the UK clinical population was studied whereby OUD typically arises from heroin; so the findings may not generalise to contexts in which there is prevalent use of highly-potent opioid analgesics, such as illicitly manufactured fentanyl, nitazines, and their analogues. However, an encouraging recent experimental study has demonstrated that BUP (at plasma levels of 2 ng/mL and higher) is protective against fentanyl-induced respiratory depression^63^

Fourth, the cost utility analysis findings were subject to uncertainty relating to missing data and uncertainty in the costs used for societal perspective. A planned analysis of data utilising registry data (e.g., healthcare utilisation recorded by Hospital Episode Statistics and criminal convictions recorded by the Police National Computer databases) should reduce parameter uncertainty and limit the potential impact of recall bias.

In this first superiority trial of BUP-XR, we conclude that in the intention-to-treat population, monthly BUP-XR has superior effectiveness relative to SoC in reducing opioid use. This is also reflected in longer treatment retention, more likelihood of early OUD remission, reduced craving, and better patient-reported and clinician-reported outcomes. BUP-XR was not cost-effective in a base case cost-utility analysis using the societal perspective; but it was cost-effective (dominant) among participants with more severe OUD, or those whose current treatment episode is longer than 28 days. Further trials are needed to evaluate if BUP-XR is associated with better clinical and health economic outcomes over the longer term.

## Contributors

JM, MK, LM, CM and ZH designed the study. ZH, RE and JM devised the SAP and DH and WH devised the HEAP. JK, CM and JB developed the data management procedure, and JB and NK developed the data capture documents. ZH, RE, DH and WH analysed the data. JB, ZH, RE, DH and JM directly accessed the data and verified the underlying data reported.

## Data sharing statement

Deidentified patient data will not be made available unless a proposal to access, that is submitted to the corresponding author from researchers employed by non-commercial organisations, is judged by the sponsor, research partners and Health Research Authority, to have sufficient scientific merit and value in the context of the study publication plan.

## Declaration of interests

In the past three years, JM declares research grants for the following clinical trials: the National Institute for Health Research (NIHR; trial of behavioural reinforcement of acamprosate for alcohol use disorder [AUD]; sponsor: King’s College London [KCL]); Indivior (the present trial of extended-release pharmacotherapy for opioid use disorder; sponsor: KCL and South London & Maudsley NHS Trust); and Beckley PsyTech (phase IIa trial of 5-MeO-DMT for alcohol use disorder [AUD]; sponsor: Beckley PsyTech). He is the senior academic advisor for the Office for Health Improvement and Disparities, English Department of Health and Social Care, and a clinical academic consultant for the US National Institute on Drug Abuse, Clinic for Clinical Trials Network. JM declares honoraria and travel support from PCM Scientific, OPEN Health, and Indivior to contribute to scientific and educational meetings. He holds no stocks in any company.

In the past three years, MK declares research grants for the following clinical trials: Indivior (the present trial of extended-release pharmacotherapy for opioid use disorder; sponsor: KCL and South London & Maudsley NHS Trust); and Beckley PsyTech (phase II trial of 5-MeO-DMT for AUD; sponsor: Beckley PsyTech). He is the national clinical advisor for the Office for Health Improvement and Disparities, English Department of Health and Social Care.

In the past three years, FC declares her membership of the NHS Scotland advisory group for a position statement on the use of long-acting injectable buprenorphine for opioid substitution therapy (Health Improvement Scotland (SIGN publication 165; https://www.sign.ac.uk/media/1947/buprenorphine-position-statement-sign-165.pdf).

In the past three years, ED declares two professional roles: Presidency of the Society for the Study of Addiction (unpaid), and UK Government National Recovery Champion for drugs (secondment from his university position).

In the past three years, LM declares research grants for the following studies: NIHR (realist evaluation of services for people with co-occurring mental health and substance use); Indivior (the present trial of extended-release pharmacotherapy for opioid use disorder; sponsor: KCL and South London & Maudsley NHS Trust); and Beckley PsyTech (phase II trial of 5-MeO-DMT for AUD; sponsor: Beckley PsyTech). He declares receipt of author royalties for a clinical textbook on CBT and addiction (published by Wiley). He has received consulting fees from the British Association of Counselling and Psychotherapy for work on counselling competencies. He is a member of the British Psychological Society’s Faculty of Addiction Committee, and he has a clinical psychology secondment at the Office for Health Improvement and Disparities, English Department of Health and Social Care.

All other authors have no interests to declare.

## Appendix ASupplementary data

Appendix I and II, Supplementary Figs. S1.1, S2.2–S2.6, and Tables S1.1–S1.4, S2.1–S2.9
