# Supplementary material for: Superiority and cost-effectiveness of monthly extended-release buprenorphine versus daily standard of care medication: a pragmatic, parallel-group, open-label, multicentre, randomised, controlled, phase 3 trial
Source: eClinicalMedicine. 2023 Nov 17;66:102311. doi: 10.1016/j.eclinm.2023.102311 (PMC10692661; doi:10.1016/j.eclinm.2023.102311)
Supplement: Appendix I and II, Supplementary Figs. S1.1, S2.2–S2.6, and Tables S1.1–S1.4, S2.1–S2.9 [file mmc1.docx]

**Superiority and cost-effectiveness of monthly extended-release buprenorphine versus daily standard of care medication: a pragmatic, parallel-group, open-label, multicentre, randomised, controlled, phase 3 trial**

John Marsden, Mike Kelleher, Eilish Gilvarry, Luke Mitcheson, Jatinder Bisla, Angela Cape, Fiona, Cowden, Edward Day, Jonathan Dewhurst, Rachel Evans, Will Hardy, Andrea Hearn, Joanna Kelly, Natalie Lowry, Martin McCusker, Caroline Murphy, Robert Murray, Tracey Myton, Sophie Quarshie, Rob Vanderwaal, April Wareham, Dyfrig Hughes, Zoë Hoare

| **Tables** | **PAGE** |
| --- | --- |
| **Table S1.1**: Dosing schedule for BUP-XR in the study | 2 |
| **Table S1.2**: Use of cocaine and benzodiazepines (full analysis set) | 3 |
| **Table S1.3**: Observed responses to CGI-S/CGI-I and PRO-S/PRO-I by treatment group | 4 |
| **Table S1.4**: Safety – adverse events: all types (full analysis set) | 5 |
| **Table S2.1**: Unit costs relating to resources measured with the ADSUS and KCF (2021 price) | 10 |
| **Table S2.2**: Trial medication unit costs (GBP), taken from the Drug Tariff Part VIIIA and dm+d. | 13 |
| **Table S2.3**: Standard care dispensing costs (GBP)…. | 15 |
| **Table S2.4**: Frequency (%) of missing data by outcome, trial group, and timepoint. | 20 |
| **Table S2.5**: Observed resource use frequency by group and timepoint. | 22 |
| **Table S2.6**: Disaggregated and total unadjusted mean (95% CR) resource use costs… | 24 |
| **Table S2.7**: Utilities based on responses to the EQ-5D-5L and EQ-VAS thermometer… | 26 |
| **Table S2.8**: Summary of cost-utility analyses based on bootstrapped and imputed data… | 30 |
| **Table S2.9:** Results of subgroup analyses, based on bootstrapped and imputed data… | 32 |

| **Figures** | **PAGE** |
| --- | --- |
| **Figure S1.1**: Number and dose of BUP-XR treatment by protocol | 6 |
| **Figure S2.1**: Drug Tariff prices for buprenorphine tablets | 15 |
| **Figure S2.2**: Distribution of participants’ responses to each EQ-5D attribute… | 25 |
| **Figure S2.3**: Cost-effectiveness plane showing the bootstrapped mean differences… | 27 |
| **Figure S2.4**: Cost-effectiveness acceptability curve for base case analysis… | 27 |
| **Figure S2.5**: Tornado plot of univariate sensitivity analyses... | 28 |
| **Figure S2.6**: Cost-effectiveness acceptability curves for each analysis… | 31 |

**APPENDIX I**

**Table S1.1**. Dosing schedule for BUP-XR in the study

| **Dose** | **Scheduled day** | **Visit** | **Window (day)** | **Dose** |
| --- | --- | --- | --- | --- |
| 1 | 1 | Week 1 | 1-7 | 300mg (loading) |
| 2 | 28 | Week 4 | 21-42 | 300mg (loading) |
| 3 | 56 | Week 8 | 54-70 | 100mg or 300mg * |
| 4 | 84 | Week 12 | 82-98 | 100mg or 300mg * |
| 5 | 112 | Week 16 | 110-126 | 100mg or 300mg * |
| 6 | 140 | Week 20 | 138-168 | 100mg or 300mg * |
| 7> | 168> | Week 24> (every 28 days) | Up to 42 days > previous dose | 100mg or 300mg * |

BUP-XR, extended-release, injectable buprenorphine.

* If the participant missed a scheduled maintenance injection no adjustment in dose was required, if they receive this dose within 60 days of their last injection. If the participant did not receive an injection within 60 days of their last injection, but wanted maintenance treatment, they were offered BUP-SL or MET according to assessment and preference following standard practice.

**Table S1.2**: Use of cocaine and benzodiazepines (full analysis set)

|  | **BUP-SL/MET** | | | **BUP-XR** | | |
| --- | --- | --- | --- | --- | --- | --- |
| **Study point** | **n** | **Cocaine** | **Benzodiazepines** | **n** | **Cocaine** | **Benzodiazepines** |
| Screening | 156 | 7·31 (10·64) | 3·01 (7·62) | 158 ***** | 5·63 (0·09) | 1·79 (5·84) |
| Weeks 1–4 | 125 | 6·48 (8·84) | 2·93 (7·20) | 153 | 4·57 (7·07) | 1·95 (5·57) |
| Weeks 5–8 | 123 | 6·55 (9·07) | 2·79 (6·60) | 145 | 4·21 (6·63) | 2·32 (6·76) |
| Weeks 9–12 | 116 | 6·47 (9·60) | 2·46 (6·68) | 142 | 4·32 (6·56) | 2·21 (6·22) |
| Weeks 13–16 | 109 | 5·92 (9·17) | 2·45 (6·58) | 139 | 4·15 (6·60) | 2·25 (6·15) |
| Weeks 17–20 | 105 | 5·70 (8·87) | 2·24 (6·27) | 131 | 4·58 (6·96) | 2·21 (6·13) |
| Weeks 21–24 | 70 | 4·23 (7·80) | 2·41 (7·15) | 108 | 4·43 (6·54) | 2·43 (7·02) |

Data is number of participants providing data on days used drug in past 28 days (standard deviation);

* missing data for 1 participant for cocaine at screening (n=157).

**Table S1.3**: Observed responses to CGI-S/CGI-I and PRO-S/PRO-I by treatment group

|  | **Baseline** | | |  | **24-week follow-up** | | |
| --- | --- | --- | --- | --- | --- | --- | --- |
| **CGI-S** | **SoC**  **(n=156)** | **BUP-XR**  **(n=158)** | **Overall**  **(n=314)** | **CGI-I** | **SoC**  **(n=156)** | **BUP-XR**  **(n=158)** | **Overall**  **(n=314)** |
| Extremely mild | 29 (18·6) | 37 (23·4) | 66 (21·0) | Very much improved | 9 (5·8) | 48 (30·4) | 57 (18·2) |
| Very mild | 16 (10·3) | 20 (12·7) | 36 (11·5) | Much improved | 21 (13·5) | 51 (32·3) | 72 (22·9) |
| Mild | 45 (28·9) | 33 (20·9) | 78 (24·8) | Minimally improved | 15 (9·6) | 13 (8·2) | 28 (8·9) |
| Moderate | 25 (16·0) | 30 (19·0) | 55 (17·5) | No change | 34 (21·8) | 9 (5·7) | 43 (13·7) |
| Severe | 18 (11·5) | 21 (13·3) | 39 (12·4) | Minimally worse | - | 1 (0·6) | 1 (0·3) |
| Very severe | 10 (6·4) | 7 (4·4) | 17 (5·4) | Much worse | 2 (1·3) | 1 (0·6) | 3 (1·0) |
| Extremely severe | 13 (8·3) | 10 (6·3) | 23 (7·3) | Very much worse | 1 (0·6) | 1 (0·6) | 2 (0·6) |
| Missing | - | - |  | Missing | 74 (47·4) | 34 (21·5) | 108 (34·4) |
| **PRO-S** |  |  |  |  |  |  |  |
| Extremely mild | 59 (37·8) | 59 (37·3) | 118 (337·6) | Very much improved | 20 (12·8) | 81 (51·3) | 101 (32·2) |
| Very mild | 17 (10·9) | 14 (8·9) | 31 (9·9) | Much improved | 26 (16·7) | 23 (14·6) | 49 (15·6) |
| Mild | 27 (17·3) | 26 (16·5) | 53 (16·9) | Minimally improved | 10 (6·4) | 5 (3·2) | 15 (4·8) |
| Moderate | 16 (10·3) | 32 (20·3) | 48 (15·3) | No change | 26 (16·7) | 11 (7·0) | 37 (11·8) |
| Severe | 21 (13·5) | 15 (9·5) | 36 (11·5) | Minimally worse | - | 1 (0·6) | 1 (0·3) |
| Very severe | 8 (5·1) | 6 (3·8) | 14 (4·5) | Much worse | - | - | - |
| Extremely severe | 8 (5·1) | 5 (3·2) | 13 (4·1) | Very much worse | 2 (1·3) | - | 2 (0·6) |
| Missing | - | 1 (0·6) | 1 (0·3) | Missing | 72 (46·2) | 37 (23·4) | 109 (34·7) |

Figures in parenthesis are percentage. Means are unadjusted

BUP-XR, extended-release buprenorphine; GSI-S, Global Severity Index-Severity; GSI-I, Global Severity Index-Improvement;

PRO-S, Patient-Rated Outcome–Severity; PRO-S, Patient-Rated Outcome–Improvement; SoC, standard-of-care.

**Table S1.4**: Safety – adverse events: all types (full analysis set)

| **Adverse event** | **BUP-SL/MET (n=156)** | **BUP-XR (n=158)** |
| --- | --- | --- |
| **Treatment-emergent adverse events** |  |  |
| Number of participants with one or more adverse event | 67 (42·9) | 128 (81·0) |
| Total number of adverse events | 133 | 450 |
| **Types of adverse event *** |  |  |
| Drug administration (pain and pruritis) | 4 (3·0) | 121 (26·9) |
| Gastrointestinal disorders | 13 (9·8) | 61 (13·6) |
| Nervous system disorders | 2 (1·5) | 42 (9·3) |
| Psychiatric disorders | 20 (15·0) | 32 (7·1) |
| Infections and infestations | 38 (28·6) | 34 (7·6) |
| Musculoskeletal and connective tissue disorders | 9 (6·8) | 35 (7·8) |
| Injury, poisoning, and procedural complications | 13 (9·7) | 22 (4·9) |
| Skin and subcutaneous tissue disorders | 5 (3·8) | 24 (5·3) |
| Neoplasms benign, malignant and unspecified | 1 (0·8) | 1 (0·2) |
| Blood and lymphatic system disorders | 3 (2·3) | 2 (0·4) |
| Immune system disorders | 0 (-) | 1 (0·2) |
| Endocrine disorders | 0 (-) | 5 (1·1) |
| Metabolism and nutrition disorders | 0 (-) | 2 (0·4) |
| Eye disorders | 0 (-) | 4 (0·9) |
| Ear and labyrinth disorders | 0 (-) | 4 (0·9) |
| Cardiac disorders | 1 (0·8) | 1 (0·2) |
| Vascular disorders | 1 (0·8) | 0 (-) |
| Respiratory, thoracic and mediastinal | 6 (4·5) | 16 (3·6) |
| Hepatobiliary disorders | 3 (2·3) | 14 (3·1) |
| Renal and urinary disorders | 1 (0·8) | 9 (2·0) |
| Reproductive system and breast disorders | 0 (-) | 2 (0·4) |
| Surgical and medical procedures | 4 (3·0) | 5 (1·1) |
| Social circumstances | 6 (4·5) | 10 (2·2) |

Data are number (%).

Main report shows drug poisoning (overdose) adverse events

BUP-SL, oral buprenorphine;

MET, oral methadone;

BUP-XR, injectable buprenorphine.

**Figure S1.1**: Number and dose of BUP-XR treatment by protocol (n=158)

| 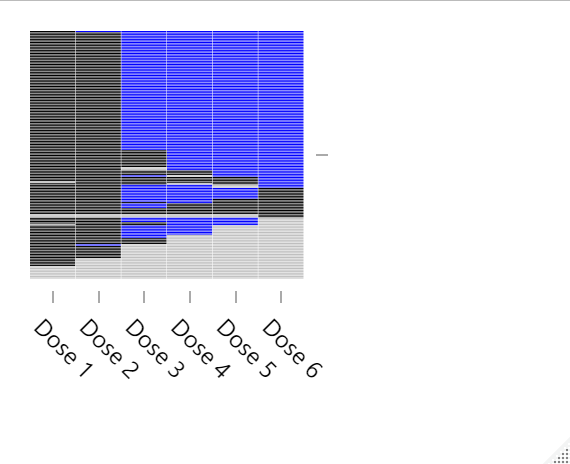 | \|  \| 300mg \| \| --- \| --- \| \|  \|  \| \|  \| 100mg \| \|  \|  \| \|  \| No dose/missed dose \|   Chart colour coding: |
| --- | --- | --- | --- | --- | --- | --- | --- | --- | --- | --- | --- |

|  |  |  |
| --- | --- | --- |

Each row represents a participant. Data is ranked by available data.

Protocol is 6 doses: dose 1 and 2 is 300mg; dose 4–6 is 100mg.

After loading (dose 1), protocol gives flexibility (i.e. 6 x 300mg; 1 x 300mg and 5 x 100mg; and combination – according to symptom control and patient preference).

Note:

Loading dose interval between dose 1 and dose 2 was:

Day 20 (1 [0.63%] of 158 – reported in manuscript as a protocol deviation

Day 21–25 (91 [57.6%] of 158)

Day 26–41 (49 [31.0%] of 158)

Missed first or second or both loading doses (17 [10.8%] of 158)

**APPENDIX II**

## 2.1 Methods

### 2.1.1 Overview

A cost-utility analysis was conducted, adopting a societal cost perspective in England, to estimate whether subcutaneously-administered, extended-release buprenorphine (BUP-XR) represents a cost-effective alternative to standard care oral medication for the treatment of opioid-use disorder. A within-trial analysis was performed using individual, patient-level data from the EXPO trial over the first 24 weeks post-randomisation. The primary outcome was the incremental cost-effectiveness ratio (ICER), expressed as costs per quality-adjusted life years (QALY) gained, calculated according to the preregistered Health Economics Analysis Plan.^1^

The health economic analysis was carried out in RStudio (see [2.4 Package citations](#package-citations) for version and package information) and reported according to the CHEERS statement.^2^

### 2.1.2 Data sources

#### 2.1.2.1 Resource use

Participants’ use of resource was considered in eight broad categories: i) accommodation, ii) productivity loss, iii) secondary care (accident and emergency, inpatient, and outpatient), iv) community services, v) criminal justice, vi) addiction keyworker, vii) use of trial medication, and viii) use of other medications. The measurement of resource-use was based on data collected as part of the trial. The first five categories were measured using the Adult Service Use Schedule (ADSUS), at baseline, week-12, and week-24. The ADSUS has been used in several previous studies of substance misuse interventions.^3–5^ Addiction key worker time was measured using the Addiction Clinic Keyworker Contact Form (KCF), at weeks 4, 12, 24. The KCF was developed by the trial research team for use in the EXPO trial. Use of trial medication was recorded on trial medication logs, continuously for oral buprenorphine or methadone and at weeks 1, 4, 8, 12, 16, and 20 for BUP-XR; concomitant medications were recorded continuously and reviewed at weeks 4, 12, and 24.

The ADSUS and KCF are paper-based forms that were completed by researchers during face-to-face interviews with participants before being entered into the electronic trial database. An exception to this was from 02/04/2020-12/06/2020 where interviews were conducted via telephone due to COVID.

All resource-use was measured irrespective of whether they were related to opioid-use disorder.^6^

##### 2.1.2.1.1 ADSUS

In all cases, participants were asked to report their use of primary and secondary care, social services, and criminal justice for the three-month period prior to completing the ADSUS. Where relevant, questions in the ADSUS contain free-text sections, which allowed participants to record any resource-use which would not otherwise be captured based on the pre-defined choices. Any relevant resources were extracted.

##### 2.1.2.1.2 KCF

The KCF asks participants about the number of “short (approximately less than 30 minutes)” and “longer (approximately 30 minutes or more)” conversations that participants had with their keyworker in the month prior to completing the questionnaire. This required the following assumptions to be made when estimating costs:

1. That short conversations lasted 15 minutes
2. That longer conversations lasted 45 minutes
3. That the number of keyworker conversations in weeks 5-8 (where no data were collected) was the same as between weeks 9-12 (where data were collected at week 12). Accordingly, we multiplied the reported resource use at week 12 by 2 to cover the period between weeks 5 and 12.
4. That the number of keyworker conversations in weeks 13-20 (where no data were collected) was the same as between weeks 21-24 (where data were collected at week 24). Accordingly, we multiplied the reported resource use at week 24 by 3 to cover the period between weeks 13-24.

##### 2.1.2.1.3 Trial medication, monitoring, and administration

For the standard care group, medication, dose, start date, end date, and reasons for changes were recorded in the oral buprenorphine/methadone medication record. In addition to trial medication use, these data, combined with within-trial survey data, were utilised to estimate: i) the number of times that a participant restarted oral medication upon prior discontinuation, ii) the time required to monitor participants receiving oral treatment, and iii) pharmacy dispensing costs.

A restart was defined as a new instance of medication use that was more than three days after the end of the previous instance and was assumed to require 105 minutes of nurse prescriber time (AfC Band 8a), based on one 60-minute assessment and an additional 45-minute follow-up during the first week of treatment to oversee dose increase titrations, liaise with clinical team and pharmacy, and write up notes. For each complete month of oral treatment, we assumed one 15-minute session with a nurse prescriber (AfC Band 8a) to check and adjust the treatment dose.

Dispensing oral methadone and buprenorphine attract specific costs based on the number of doses, number of pick-ups from the pharmacy, and the number of doses consumed under supervision. Patient level data were not widely available to reliably estimate individual pharmacy contacts; therefore, a survey of trial sites (n = 5) was carried out to estimate mean frequency of medication pick-ups and supervised doses for oral methadone and buprenorphine products. Based on the mean of survey responses, we assumed that for each 14-day period, patients would pick-up their medication 8·4 times and would consume 36·7% of doses under supervision. To reflect different practices, scenario analyses were performed, in which i) medication was picked up six times per week, with supervised consumption at each visit (i.e., 12 pick-ups and supervised doses in a 14-day period), and ii) medication was picked up once per fortnight and no doses were consumed under supervision.

We assumed that BUP-XR was administered by a nurse prescriber (AfC Band 8a) and that this took 16 minutes, based on the 15 minutes required for the product to reach room temperature once removed from refrigerated storage and 1 minute for administration.^7^

Liver function tests (LFT) were administered to all patients at baseline, but only to BUP-XR participants during the trial. However, it is recommended that all patients receiving buprenorphine therapy have their liver function monitored throughout treatment.^8^ Therefore, we assumed that any patient receiving buprenorphine at week-12 or week-24 would also receive an LFT.

Trial medication use and monitoring was collected by researchers from patients’ medical records and entered recorded on paper forms before being entered into the electronic trial database.

##### 2.1.2.1.4 Concomitant medications

Participants’ use of concomitant medicines was recorded throughout the trial and was gathered from patients’ medical records and by researchers asking the patients to report medication use. These data were then recorded in the concomitant medication log on the electronic trial database. The adverse events log and medical history form were used to fill missing values where possible. Where start dates were missing, we assumed that treatment began >90 days before randomisation. Where end dates were missing and, at the last visit, treatment was ongoing, we assumed that treatment continued beyond the end of the trial. In cases where any concomitant medication began before randomisation but was not ongoing and no end-date recorded, we assumed that treatment ended at randomisation. Where the treatment period overlapped randomisation, the period was split into pre- and post-randomisation periods to apportion the resource-use to the respective periods. If the frequency of medication use was missing, we assumed the weighted mean frequency of other participants’ use of that medication, and when that was not available, we assumed the recommended dose regimen listed in the British National Formulary (BNF).^9^ COVID-19 vaccinations were excluded from the analysis.

#### 2.1.2.2 Unit costs

Resource-use was valued in monetary terms (£GBP) at the time of analysis (cost year: 2020-2021) and were not discounted as the trial follow-up was less than one-year. Adjustments were made for inflation, when necessary, using the Hospital & Community Health Services (HCHS) Index according to the current version of the compendium of Personal Social Services Research Unit (PSSRU) Unit Costs of Health and Social Care.^10^ Total costs for resource-use were calculated by multiplying the unit cost per item by the recorded number of times that each resource was used.

Hospital inpatient stays, day case, and outpatient attendances were costed according to National Reference Costs.^11^ Primary health care, community care, housing, and criminal justice unit costs were sourced from the PSSRU Unit Costs of Health and Social Care,^10^ Unit Costs in Criminal Justice,^12^ Home Office costs of crime reports,^13–15^ and supplemented with data from published literature or local estimates when necessary. We truncated the frequency of crime at a maximum of five instances, in accordance with the methods used to estimate the unit costs of crime.^16^

Costs due to productivity losses were estimated using the human capital method,^17^ multiplying the days absent from work due to illness with average annual earnings data matched by sex, obtained from the Office for National Annual Survey of Hours and Earnings.^18^ Addiction keyworker costs were based on the results of a previous trial.^5^ [Table S2.1](#tbl-adsus-unit-costs) presents the unit costs relating to resources measured using the ADSUS and KCF.

Table S2.1: Unit costs (GBP) relating to resources measured with the ADSUS and KCF (2021 price)

| Description | Unit cost | Notes | Source |
| --- | --- | --- | --- |
| **Societal perspective** | | | |
| Staffed accommodation | 950·00 | Per week | [10] |
| Homeless: hostel, shelter, or refuge | 264·96 | Per week | [93] |
| Prison | 124·79 | Per prisoner night (overall resource/average population/year) | [12] |
| Probation officer | 236·70 | Per contact hour | [12] |
| Solicitor or other legal representative (for any reason, e.g. mental health, criminal, or civil) | 244·98 | Per hour | [13] |
| Legal aid | 50·11 | Per hour | [13] |
| Burglary in a dwelling | 6,603·29 | Per instance | [13] |
| Burglary not in a dwelling | 17,215·32 | Per instance | [13] |
| Robbery of personal property | 12,605·26 | Per instance | [13] |
| Theft of a vehicle | 11,458·32 | Per instance | [13] |
| Theft from a vehicle | 968·78 | Per instance | [13] |
| Theft from a person | 1,536·68 | Per instance | [13] |
| Theft from a shop | 1,080·13 | Per instance. Commercial theft. | [13] |
| Criminal damage to a dwelling | 1,503·28 | Per instance | [13] |
| Common assault | 6,603·29 | Per instance. Violence without injury. | [13] |
| Harassment | 6,603·29 | Per instance. Assume same as common assault - violence without injury. | [13] |
| Cheque and credit card fraud | 1,436·47 | Per instance. Fraud. | [13] |
| Violence with injury | 15,645·23 | Per instance | [13] |
| Violence without injury | 6,603·29 | Per instance | [13] |
| Theft or unauthorized taking of a bicycle | 590·36 | Per instance | [15] |
| Median gross weekly earning for full-time females | 558·10 | Median (mean not available) | [18] |
| Median gross weekly earning for full-time males | 651·60 | Median (mean not available) | [18] |
| Begging | 0·00 | Zero rated as per ARC trial | [5] |
| Police custody | 100·50 | Per night | [87] |
| Drug dealing | 5,172·33 | Per instance | [87] |
| Drug growing | 5,172·33 | Per instance | [87] |
| Police (as either victim or perpetrator of crime) | 64·32 | Per hour | [91] |
| Handling stolen goods | 3,557·13 | Per instance. Miscellaneous crimes against society. | [92] |
| Drunk and disorderly | 1,401·29 | Per instance. Public order offence. | [92] |
| Sex work | 754·54 | Per instance. Summary non-motoring offence. | [92] |
| Driving whilst disqualified | 215·58 | Per instance. Summary motoring. | [92] |
| Speeding | 215·58 | Per instance. Summary motoring. | [92] |
| Trespassing | 754·54 | Per instance. Summary non-motoring offence. | [92] |
| Fare evasion | 754·54 | Per instance. Summary non-motoring offence. | [92] |
| Breach of the peace | 1,401·29 | Per instance. Public order offence. | [92] |
| Homeless: living on the streets | 0·00 | Per week |  |
| Homeless: living with friends or relatives | 0·00 | Per week |  |
| **PSS perspective** | | | |
| Social worker/child and family support worker | 0·87 | Per minute | [10] |
| Home help/support worker | 0·55 | Per minute. Assume weekday hour provided for social services. | [10] |
| Day centre | 0·16 | Per minute. Assume for mental health support. | [10] |
| Drop in centre | 0·16 | Per minute. Assume same as day centre. | [10] |
| Art/music/drama therapy | 0·78 | Per minute. Assume same as community OT. | [10] |
| Housing support worker | 0·58 | Per minute. Assume social work assistant. | [10] |
| Advice service (e.g. citizen's advice bureau) | 0·58 | Per minute. Assume social work assistant. | [10] |
| Self-help groups (e.g. AA) | 0·58 | Per minute. Assume social work assistant. | [10] |
| Helpline (e.g. Samaritans) | 4·37 | Per call | [94] |
| **NHS perspective** | | | |
| Inpatient (long stay) | 3,627·00 | Five or more days | [10] |
| Inpatient (short stay) | 827·00 | Less than five days | [10] |
| Outpatient | 137·00 |  | [10] |
| Ambulance | 134·00 | Average of all | [10] |
| GP – practice | 4·30 | Per minute | [10] |
| GP – telephone | 4·13 | Per minute | [10] |
| Practice nurse (in GP surgery) | 0·73 | Per minute | [10] |
| Health visitor/district nurse | 0·92 | Per minute. Band 6 nurse | [10] |
| Occupational therapist | 0·78 | Per minute | [10] |
| Counsellor | 0·90 | Per minute. Band 6 | [10] |
| Psychotherapist | 1·08 | Per minute. Assumed same as clinical psychologist, Band 7 | [10] |
| Family therapist | 0·90 | Per minute. Assume same as counsellor, Band 6 | [10] |
| Drug and alcohol counsellor/support worker outside EXPO clinic | 1·25 | Per minute | [10] |
| Depot clinic outside EXPO clinic | 1·10 | Per minute. Assume mental health nurse, Band 7. | [10] |
| Dentist | 3·38 | Per minute. Assume providing performer | [10] |
| Dietician | 0·65 | Per minute. Assume Band 5 | [10] |
| Consultant | 2·05 | Per minute. Assume medical consultant. | [10] |
| Optician | 0·87 | Per minute. Assume Band 6 | [10] |
| Nurse prescriber | 1·17 | Per minute. Assume Band 8a. | [10] |
| Community nurse | 0·92 | Per minute. Assume Band 6 community nurse. | [10] |
| Blood clinic | 0·55 | Per minute. Assume Band 4 nurse. | [10] |
| Restart costs | 122·50 | 105 minutes of a band 8a nurse prescriber's time | [10] |
| Monitoring costs | 17·50 | 15 minutes per month of band 8a nurse prescriber time to check and adjust dosages. | [10] |
| Addiction keyworker time | 1·34 | Per minute | [5] |
| Accident and emergency | 153·67 |  | [88] |
| Liver function test | 5·05 | For buprenorphine, regardless of formulation, the recommendation is: Monitor liver function; when used in opioid dependence baseline liver function test is recommended before commencing therapy, and regular liver function tests should be performed throughout treatment (BNF, 2022). Assume once every 12 weeks. | [90] |
| Smoking cessation service | 0·49 | Per minute. Include costs associated with time spent with adviser but not pharmacotherapy prescribed. | [95] |

The unit costs of trial medicines (except BUP-XR) were taken from the BNF,^9^ to reflect the prices that would be paid by the NHS as closely as possible.^19^ For branded preparations, the BNF indicative prices are derived from the NHS Dictionary of Medicines and Devices (DM+D). Drug Tariff Part VIIIA prices listed in the BNF (April 2021) were applied to generics, reflecting pharmacist reimbursement. A unit cost for BUP-XR (the intervention) was provided by the manufacturer. [Table S2.2](#tbl-trial-med-costs) presents the unit costs of trial medication.

Table S2.2: Trial medication unit costs (GBP), taken from the Drug Tariff Part VIIIA and dm+d.

| Description | Unit | Unit cost | Notes |
| --- | --- | --- | --- |
| XR-Bup | Per injection | 262·90 | Indicative unit cost provided by Indivior for primary analyses |
| XR-Bup low cost | Per injection | 239·70 | Lower indicative unit cost provided by Indivior for sensitivity analyses |
| Buprenorphine sublingual tablet 200mcg | Per tablet | 0·10 |  |
| Buprenorphine sublingual tablet 400mcg | Per tablet | 0·20 |  |
| Buprenorphine sublingual tablet 2mg | Per tablet | 0·51 |  |
| Buprenorphine sublingual tablet 8mg | Per tablet | 1·37 |  |
| Suboxone (buprenorphine + naloxone) 2mg | Per tablet | 0·91 | Generic and branded same price |
| Suboxone (buprenorphine + naloxone) 8mg | Per tablet | 2·46 | Generic and branded same price |
| Suboxone (buprenorphine + naloxone) 16mg | Per tablet | 5·44 | Generic and branded same price |
| Espranor oral lyophilisates 2mg | Per tablet | 0·91 | Generic and branded same price |
| Espranor oral lyophilisates 8mg | Per tablet | 2·72 | Generic and branded same price |
| Methadone 1mg/ml oral solution | Per millilitre | 0·01 | Assume 1mg/ml. Stronger formulations (10mg/ml or 20mg/ml) would lower this to £0.0080 per ml |
| Buprenorphine sublingual tablet 200mcg | Per tablet | 0·10 | February 2019 price - For high buprenorphine cost scenarios |
| Buprenorphine sublingual tablet 400mcg | Per tablet | 0·23 | February 2019 price - For high buprenorphine cost scenarios |
| Buprenorphine sublingual tablet 2mg | Per tablet | 0·95 | February 2019 price - For high buprenorphine cost scenarios |
| Buprenorphine sublingual tablet 8mg | Per tablet | 2·74 | February 2019 price - For high buprenorphine cost scenarios |
| Suboxone (buprenorphine + naloxone) 2mg | Per tablet | 0·92 | February 2019 price - For high buprenorphine cost scenarios |
| Suboxone (buprenorphine + naloxone) 8mg | Per tablet | 2·75 | February 2019 price - For high buprenorphine cost scenarios |
| Suboxone (buprenorphine + naloxone) 16mg | Per tablet | 5·44 | February 2019 price - For high buprenorphine cost scenarios |
| Buprenorphine sublingual tablet 200mcg | Per tablet | 0·10 | July 2018 price - For low buprenorphine cost scenarios |
| Buprenorphine sublingual tablet 400mcg | Per tablet | 0·23 | July 2018 price - For low buprenorphine cost scenarios |
| Buprenorphine sublingual tablet 2mg | Per tablet | 0·11 | July 2018 price - For low buprenorphine cost scenarios |
| Buprenorphine sublingual tablet 8mg | Per tablet | 0·26 | July 2018 price - For low buprenorphine cost scenarios |
| Suboxone (buprenorphine + naloxone) 2mg | Per tablet | 0·88 | July 2018 price - For low buprenorphine cost scenarios |
| Suboxone (buprenorphine + naloxone) 8mg | Per tablet | 2·72 | July 2018 price - For low buprenorphine cost scenarios |
| Suboxone (buprenorphine + naloxone) 16mg | Per tablet | 5·44 | Introduced in February 2019 and consistent since |

It is important to note that the cost of buprenorphine has fluctuated considerably since April 2018 with the costs for all seven oral products peaking in February 2019 (see [Figure S2.1](#fig-bup-price-changes)). The greatest differences were for 2mg and 8mg sublingual buprenorphine tablets, which were nearly twice as expensive in February 2019 than in April 2021.

| 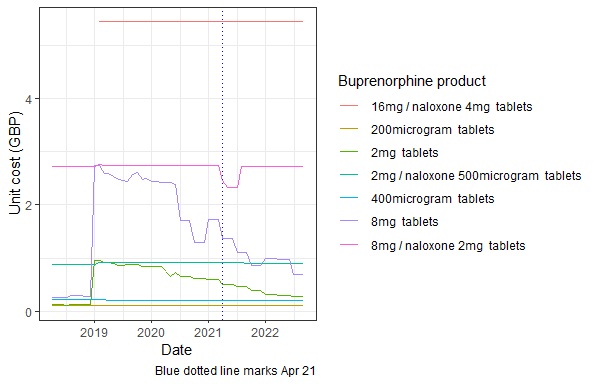  Figure S2.1: Drug Tariff prices for buprenorphine tablets |
| --- |

Fees associated with dispensing standard care were sourced from the Pharmacy Services Negotiating Committee (PSNC)^20^ and supplemented by local estimates of supervised consumption fees, which are agreed locally (see [Table S2.3](#tbl-sc-disp-costs)). The effect of oral buprenorphine price on the cost-effectiveness of BUP-XR was explored using scenario analyses.

Table S2.3: Standard care dispensing costs (GBP), based on PSNC website and supplemented with estimates from trial sites.

| Applicable fee | Methadone | Buprenorphine | Notes |
| --- | --- | --- | --- |
| Single activity fee | 1·27 | 1·27 | Per interaction or "pick-up" |
| Controlled drug fee | 1·28 | 0·43 | Per interaction or "pick-up" |
| Consumables allowance | 0·01 | 0·01 | Per interaction or "pick-up" |
| Payment for containers | 0·00 | 0·10 | Per interaction or "pick-up" |
| Item level fee | 2·50 | 0·00 | Per prescription |
| Packaged dose fee | 0·55 | 0·00 | Per additional packaged dose. Total number of separately packaged doses supplied minus total number of patient interactions |
| Supervision | 2·13 | 2·64 | Per supervised pick-up |

#### 2.1.2.3 Health utilities

The health outcome measure for the economic analysis was the quality-adjusted life year (QALY), generated from utility data measured using the EuroQol 5-dimension 5-level (EQ-5D-5L) questionnaire.^21^

The EQ-5D-5L descriptive system includes five dimensions, relating to mobility, self-care, usual activities, pain and discomfort and anxiety/depression. Each dimension is scored from 1 (no problems) to 5 (extreme problems).

All economic outcome measures were completed during the baseline visit, the week-12 visit, and the week-24 visit. Responses were then converted into a utility score using the NICE recommended tariff values,^19^ which is currently the EQ-5D-5L crosswalk to the EQ-5D-3L UK set.^22^ The EuroQol Visual Analogue Scale (EQ-VAS) provides a direct valuation of respondents’ current state of health (measured on a scale of 0 to 100, representing worst to best imaginable health) and was used as a secondary health outcome measure after dividing the reported values by 100. QALY profiles were derived from these utilities, estimated based on the area under the curve (AUC) assuming the trapezoidal rule using all available data.

QALYs were not discounted as the trial follow-up period was less than one year.

### 2.1.3 Missing data

Data were examined for missingness, through inspection of missingness patterns and proportions, to inform the methods for handling missing data based on likely mechanisms.^23^ Missing cost and utility data were imputed using multiple imputation with chained equations (MICE).^24^ If data are not missing at random, complete case analyses can lead to substantive bias, that may change cost-effectiveness decisions.^23^ Multiple imputation with chained equations provides unbiased results, assuming that data are missing at random.^23,24^

To maximise data use, costs and utilities were imputed at each timepoint they were measured. Imputation models used treatment group, participant’s last visit and data collected at previous time points (including baseline and randomisation data) as predictors but omitted data from future timepoints, to maximise data-use, whilst reducing the burden on the imputation models and to preserve randomisation.^25^ The imputation models included trial group as a covariate, rather than imputing trial arms separately (which may produce biased estimates).^26^ This was done as the high proportion of missing data in the standard care group (>50% for some items, see [Table S2.4](#tbl-missing-data-summary)) prevented disaggregated cost data being imputed in the bootstrapped data. Using aggregated cost data would have reduced the number of variables included in the imputation models, therefore reducing the plausibility of the assumption that data were missing at random.

Measured variables were imputed using predictive mean matching, and variables derived from measured variables (e.g., QALYs, total trial costs) were imputed using passive imputation, to retain the relationships between the variable of interest and those it is derived from.^25^ There was one exception to this, criminal justice costs, which were aggregated by timepoint prior to imputation due to issues of model convergence when included separately.

When bootstrapping was used, imputation procedures were nested within each bootstrap, consequently, we used one imputation per bootstrap instead of basing the number of imputations on the fraction of missing information as the latter may underestimate uncertainty.^27^

### 2.1.4 Data analysis

Analyses were conducted based on the intention to treat approach, including all randomised participants. All statistical tests were two-sided, with confidence-intervals (CIs) estimated using the bias-corrected and accelerated (BCa) method and central ranges (CRs) reported at 95%.

Total costs and QALYs during the 24-week trial were calculated based on the imputed bootstrapped data, with summary statistics generated by randomised treatment group. Differences between the treatment groups were compared with reference to bootstrapped central ranges, based on 10,000 replications.

#### 2.1.4.1 Regression analyses

Total costs and QALYs (at 24 weeks) were adjusted for any imbalances in baseline costs and utilities and stratification variables (study site and drug injecting status at baseline), by including these variables in cost and utility regression models.^28,29^

Participant trial costs were modelled using a generalised linear model (GLM)^30,31^ with a log-link function and gamma probability distribution. Treatment group (binary) and baseline costs (continuous) were included as independent variables with random intercepts for trial site (five-level factor) and injecting status at baseline (binary).

The suitability of several GLM specifications was assessed using Akaike Information Criterion (AIC) values (where lower values indicate better model fit), inspection of residual error plots, and based on the ability to run a given model on the bootstrapped and imputed data. Gamma, Gaussian, and Poisson distributions, each with log and identity links were considered as candidate specifications. The gamma distribution with log link was chosen as it had the second lowest AIC value, could be run reliably on the bootstrapped and imputed data, and visual inspection of the residual error plots did not suggest that the model was unsuitable for the data. A gamma distribution with an identity link had a lower AIC value, however, it could not be run reliably on the bootstrapped and imputed data.

Participant QALYs were modelled using an GLM with an identity-link function and Gaussian probability distribution (equivalent to Ordinary Least Squares), with treatment group (binary) and baseline QALYs (continuous) as independent variables and random intercepts for trial site (five-level factor) and injecting status at baseline (binary).

#### 2.1.4.2 Deviations from analysis plans

Including random intercepts for the stratification variables in the regression analyses was a deviation from the HEAP, where it was proposed that these variables would be included as covariates in seemingly unrelated regression using ordinary least squares. This was necessary due to modelling costs with a GLM with a log link, would mean that the treatment group effect would only apply to one site and injecting status.

In the HEAP, we proposed the use of seemingly unrelated regression, using ordinary least squares. However, the distribution of residual error terms and model fit (assessed using AIC) suggested that a Gamma GLM with log link was a better fit to the data and was therefore preferred for our analyses.^32^

#### 2.1.4.3 Incremental analysis

The cost-effectiveness of BUP-XR compared to standard care was assessed by its ICER, calculated as:

$$ICER=\Delta Costs/\Delta QALYs$$

where $\Delta Costs$ is the difference in mean total costs between intervention groups ($Cost_{XR bup}-Cost_{SoC}$) and $\Delta QALYs$ is the difference in mean QALYs between intervention groups ($QALY_{XR bup}-QALY_{SoC}$).

Incremental net monetary benefits (INMB) and net health benefits (INHB) were also calculated at the £20,000 and £30,000 per QALY thresholds as:

$$INHB=\Delta QALYs-\Delta Costs/\lambda$$

and

$$INMB=\Delta QALYs\cdot\lambda-\Delta Costs$$

where $\lambda$ is the cost-effectiveness threshold.^19^

#### 2.1.4.4 Parameter uncertainty

The joint uncertainty in costs and QALYs was evaluated using a non-parametric bootstrap with 10,000 replicates of the patient-level data for the primary analysis and 5,000 replications for all other analyses. Bootstrapped samples were stratified by randomised treatment group, trial site, and injecting status at baseline. Uncertainty in the ICER was represented graphically on the cost-effectiveness plane and cost-effectiveness acceptability curve.^33^

#### 2.1.4.5 Sensitivity analyses

We conducted a range of sensitivity analyses to assess the robustness of the base-case ICER to key assumptions relating to the cost of standard care, including both medication and dispensing costs, as both can vary considerably in routine practice.

##### 2.1.4.5.1 Univariate

Several univariate sensitivity analyses were conducted to identify which variables have the strongest direct effect on the cost-effectiveness results. Specifically, we varied the following costs:

- Accommodation
- Productivity loss
- Accident and emergency
- Inpatient
- Outpatient
- Community care
- Criminal justice
- Addiction keyworker
- Trial medication (including BUP-XR)
- Concomitant medication

Each cost input was varied ±10%, and the INMB calculated and plotted in a tornado plot, which ranks variables based on the influence they have on the result. The sensitivity analysis for utilities involved estimating the cost-effectiveness based on QALYs calculated from the EQ-VAS, rather than the EQ-5D-5L.

##### 2.1.4.5.2 Scenario analyses

There is considerable variation in the management of opioid-use disorder, related to both patient and local factors.^34^ In addition, the price of oral buprenorphine has varied significantly in the last three years (see [Figure S2.1](#fig-bup-price-changes)) Therefore, it was important to consider scenarios that captured this variation.

1. Assuming that all standard care participants were taking only oral methadone and incurred costs as estimated by PSSRU in 2009. These costs include capital and revenue costs (buildings and land, equipment and durables, staff costs (including site staff and external support staff), supplies and services, and site and agency overheads) and methadone costs (prescriptions, any pharmacist dispensing fees, and any toxicology tests).
2. Assuming that the price of BUP-XR is lower (£239·70)
3. Assuming that all standard care participants received daily supervised pick-ups
4. Assuming that all standard care participants were on once fortnightly pick-ups (no supervision)
5. “Best” case - Oral buprenorphine and methadone at February 2019 prices and assuming six supervised pick-ups per week
6. “Worst” case - Oral buprenorphine and methadone at July 2018 prices and assuming one unsupervised pick-up per fortnight
7. Taking an NHS + PSS + criminal justice + accommodation cost perspective
8. Taking an NHS + PSS + criminal justice cost perspective
9. Taking an NHS + PSS cost perspective
10. Taking an NHS + PSS cost perspective (BUP-XR priced lower [£239·70])
11. Taking an NHS cost perspective

#### 2.1.4.6 Subgroup analyses

A stratified cost-effectiveness analysis was conducted to investigate how cost effectiveness varied by subgroup.^35,36^ We considered the following groups (based on baseline questionnaire responses):

- Using cocaine (Yes/No)
- Length of time in treatment (less than one month/one month or longer)
- Benzodiazepine use past month to admission (Yes/No)
- CGI-S (Extremely mild-Mild vs. Moderate-Extremely severe)

To estimate the cost-effectiveness of BUP-XR within each subgroup, patient level NMB was calculated at WTPs of £0, £20k, and £30k per QALY gained. These NMBs were then used as the dependent variables in separate OLS regressions, which included baseline costs and utilities, site, trial arm, each of the subgroups, and the one-way interactions between each subgroup and trial arm as independent variables. The resulting coefficients were then used to estimate the INMB (at £20k and £30k per QALY) and incremental costs and QALYs associated with BUP-XR in each subgroup. This also allowed the calculation of ICERs for each subgroup. This was done in accordance with the methods described by Hoch et al.^35^ for conducting a stratified cost-effectiveness analysis.

## 2.2 Results

### 2.2.1 Data completeness

Data were available for 314 participants, relating to 156 participants randomised to standard care and 158 randomised to extended-release buprenorphine. A breakdown of missing data by treatment group and outcome is provided in [Table S2.4](#tbl-missing-data-summary). There was a higher proportion of missing data in the standard care group, resulting from the higher rate of drop-out from the study (45·51% at 24 weeks in the standard care group vs. 22·15% at 24 weeks in the BUP-XR group).

Table S2.4: Frequency (%) of missing data by outcome, trial group, and timepoint.

| Variable | SoC (n = 156) | BUP-XR (n = 158) |
| --- | --- | --- |
| **Baseline** | | |
| Site | 0 (0·00) | 0 (0·00) |
| Sex | 0 (0·00) | 0 (0·00) |
| Age | 0 (0·00) | 0 (0·00) |
| Ethnicity == White | 0 (0·00) | 0 (0·00) |
| Age first received opioid substitution therapy | 0 (0·00) | 1 (0·63) |
| Current episode > 28 days | 0 (0·00) | 0 (0·00) |
| Injecting status | 0 (0·00) | 0 (0·00) |
| Oral buprenorphine/methadone preference | 1 (0·64) | 0 (0·00) |
| Using heroin at | 1 (0·64) | 1 (0·63) |
| Using cocaine at | 1 (0·64) | 1 (0·63) |
| Using benzodiazepines at | 1 (0·64) | 1 (0·63) |
| Severity rating on CGI at | 0 (0·00) | 0 (0·00) |
| Accommodation | 0 (0·00) | 1 (0·63) |
| Productivity loss | 1 (0·64) | 1 (0·63) |
| Accident and emergency | 0 (0·00) | 1 (0·63) |
| Inpatient | 1 (0·64) | 2 (1·27) |
| Outpatient | 1 (0·64) | 1 (0·63) |
| Community care (NHS + PSS) | 1 (0·64) | 1 (0·63) |
| Custody | 2 (1·28) | 1 (0·63) |
| CJ professional | 1 (0·64) | 1 (0·63) |
| Victim of crime | 2 (1·28) | 1 (0·63) |
| Crime committed | 2 (1·28) | 1 (0·63) |
| Concomitant medication costs | 0 (0·00) | 0 (0·00) |
| EQ-5D | 1 (0·64) | 1 (0·63) |
| **Week 4** | | |
| Keyworker | 53 (33·97) | 15 (9·49) |
| **Week 12** | | |
| Accommodation | 66 (42·31) | 29 (18·35) |
| Productivity loss | 14 (8·97) | 15 (9·49) |
| Accident and emergency | 66 (42·31) | 29 (18·35) |
| Inpatient | 66 (42·31) | 29 (18·35) |
| Outpatient | 66 (42·31) | 29 (18·35) |
| Community care (NHS + PSS) | 67 (42·95) | 30 (18·99) |
| Custody | 66 (42·31) | 29 (18·35) |
| CJ professional | 66 (42·31) | 29 (18·35) |
| Victim of crime | 66 (42·31) | 29 (18·35) |
| Crime committed | 66 (42·31) | 28 (17·72) |
| Keyworker | 70 (44·87) | 30 (18·99) |
| EQ-5D | 67 (42·95) | 29 (18·35) |
| **Week 24** | | |
| Accommodation | 71 (45·51) | 37 (23·42) |
| Productivity loss | 13 (8·33) | 12 (7·59) |
| Accident and emergency | 71 (45·51) | 37 (23·42) |
| Inpatient | 71 (45·51) | 37 (23·42) |
| Outpatient | 71 (45·51) | 37 (23·42) |
| Community care (NHS + PSS) | 71 (45·51) | 37 (23·42) |
| Custody | 71 (45·51) | 37 (23·42) |
| CJ professional | 71 (45·51) | 37 (23·42) |
| Victim of crime | 71 (45·51) | 37 (23·42) |
| Crime committed | 71 (45·51) | 37 (23·42) |
| Keyworker | 71 (45·51) | 38 (24·05) |
| EQ-5D | 71 (45·51) | 37 (23·42) |
| Trial medication | 0 (0·00) | 0 (0·00) |
| Concomitant medication trial costs | 0 (0·00) | 0 (0·00) |

###

### 2.2.2 Resource use

[Table S2.5](#tbl-observed-resource-use-freq) summarises disaggregated resource use frequencies for each trial group at each timepoint.

Table S2.5: Observed resource use frequency by group and timepoint.

|  | SoC | | | BUP-XR | | |
| --- | --- | --- | --- | --- | --- | --- |
| Resource | Total | n | Mean | Total | n | Mean |
| **Baseline** | | | | | | |
| Accommodation (weeks) | 24 | 156 | 0·15 | 21 | 157 | 0·13 |
| Days off due to illness | 237 | 155 | 1·53 | 196 | 157 | 1·25 |
| Accident and emergency (contacts) | 42 | 156 | 0·27 | 41 | 157 | 0·26 |
| Accident and emergency (admitted) | 17 | 156 | 0·11 | 23 | 157 | 0·15 |
| Accident and emergency (ambulance) | 15 | 156 | 0·10 | 20 | 157 | 0·13 |
| Inpatient (nights) | 34 | 155 | 0·22 | 19 | 156 | 0·12 |
| Outpatient (appointments) | 27 | 155 | 0·17 | 82 | 157 | 0·52 |
| Community care (contacts) | 816 | 155 | 5·26 | 1181 | 157 | 7·52 |
| Custody (days) | 380 | 154 | 2·47 | 843 | 157 | 5·37 |
| CJ professional (contacts) | 295 | 155 | 1·90 | 164 | 157 | 1·04 |
| Victim of crime (number of offences) | 52 | 154 | 0·34 | 40 | 157 | 0·25 |
| Crime committed (number of offences) | 32 | 154 | 0·21 | 34 | 157 | 0·22 |
| **Week 4** | | | | | | |
| Keyworker (short) | 198 | 103 | 1·92 | 154 | 143 | 1·08 |
| Keyworker (long) | 21 | 103 | 0·20 | 37 | 143 | 0·26 |
| **Week 12** | | | | | | |
| Accommodation (weeks) | 17 | 90 | 0·19 | 13 | 129 | 0·10 |
| Days off due to illness | 30 | 142 | 0·21 | 17 | 143 | 0·12 |
| Accident and emergency (contacts) | 18 | 90 | 0·20 | 20 | 129 | 0·16 |
| Accident and emergency (admitted) | 11 | 90 | 0·12 | 4 | 129 | 0·03 |
| Accident and emergency (ambulance) | 10 | 90 | 0·11 | 8 | 129 | 0·06 |
| Inpatient (nights) | 32 | 90 | 0·36 | 3 | 129 | 0·02 |
| Outpatient (appointments) | 30 | 90 | 0·33 | 44 | 129 | 0·34 |
| Community care (contacts) | 632 | 89 | 7·10 | 919 | 128 | 7·18 |
| Custody (days) | 8 | 90 | 0·09 | 35 | 129 | 0·27 |
| CJ professional (contacts) | 83 | 90 | 0·92 | 174 | 129 | 1·35 |
| Victim of crime (number of offences) | 23 | 90 | 0·26 | 22 | 129 | 0·17 |
| Crime committed (number of offences) | 13 | 90 | 0·14 | 16 | 130 | 0·12 |
| Keyworker (short) | 123 | 86 | 1·43 | 141 | 128 | 1·10 |
| Keyworker (long) | 25 | 86 | 0·29 | 25 | 128 | 0·20 |
| **Week 24** | | | | | | |
| Accommodation (weeks) | 13 | 85 | 0·15 | 10 | 121 | 0·08 |
| Days off due to illness | 20 | 143 | 0·14 | 49 | 146 | 0·34 |
| Accident and emergency (contacts) | 17 | 85 | 0·20 | 17 | 121 | 0·14 |
| Accident and emergency (admitted) | 7 | 85 | 0·08 | 6 | 121 | 0·05 |
| Accident and emergency (ambulance) | 8 | 85 | 0·09 | 7 | 121 | 0·06 |
| Inpatient (nights) | 1 | 85 | 0·01 | 12 | 121 | 0·10 |
| Outpatient (appointments) | 19 | 85 | 0·22 | 57 | 121 | 0·47 |
| Community care (contacts) | 432 | 85 | 5·08 | 660 | 121 | 5·45 |
| Custody (days) | 16 | 85 | 0·19 | 17 | 121 | 0·14 |
| CJ professional (contacts) | 64 | 85 | 0·75 | 102 | 121 | 0·84 |
| Victim of crime (number of offences) | 40 | 85 | 0·47 | 18 | 121 | 0·15 |
| Crime committed (number of offences) | 13 | 85 | 0·15 | 17 | 121 | 0·14 |
| Keyworker (short) | 86 | 85 | 1·01 | 95 | 120 | 0·79 |
| Keyworker (long) | 16 | 85 | 0·19 | 15 | 120 | 0·12 |
| Note: Resource use at baseline relates to the three months prior to randomisation for all resources listed. Key worker frequencies relate to the prior month; total post-randomisation keyworker session can be estimated as week 4 + 2 * week 12 + 3 * week 24. All other resources listed under Weeks 12 and 24 relate to the three months prior. | | | | | | |

Based on the bootstrapped imputed data, unadjusted baseline costs were £4,138 (95% CR 2,968, 5,959) and £3,559 (95% CR 2,715, 4,611) for the standard care and BUP-XR groups respectively. [Table S2.6](#tbl-imp-resource-use-diffs-join) presents disaggregated, unadjusted mean costs for each trial group for the total 24-week period and the mean differences (with 95% CRs) based on bootstrapped (10,000 replicates) and imputed data. The majority of costs related to medication (trial and concomitant), accommodation, and being the victim of crime. With the exception of trial medication costs, there were no significant differences in individual unadjusted costs between the two treatment groups.

In the adjusted analysis, standard care was associated with a mean within-trial cost of £5,789 (95% CR 3,950, 9,322) and BUP-XR a mean within-trial cost of £6,821 (95% CR 4,887, 9,829). This corresponds to an incremental cost of £1,033 (95% CR -1,189, 3,225).

Table S2.6: Disaggregated and total unadjusted mean (95% CR) resource use costs by trial group for trial period (bootstrapped and imputed data).

| Cost | SoC | BUP-XR | Difference |
| --- | --- | --- | --- |
| Accommodation | 1,487.72 (777.98–2,463.22) | 1,123.63 (566.90–1,847.01) | -364.10 (-1,252.99–439.79) |
| Productivity loss | 46.28 (10.02–96.05) | 48.75 (5.77–114.65) | 2.46 (-63.55–77.27) |
| Accident & Emergency | 100.09 (42.98–184.68) | 77.81 (44.54–118.73) | -22.28 (-104.47–41.99) |
| Inpatient | 112.05 (26.51–249.62) | 79.56 (20.94–165.10) | -32.49 (-149.05–62.87) |
| Outpatient | 80.05 (39.52–136.12) | 100.84 (44.22–190.76) | 20.78 (-54.18–115.27) |
| Community services | 247.47 (173.66–354.11) | 198.85 (144.19–274.42) | -48.62 (-141.52–31.62) |
| Custody | 42.40 (12.89–96.19) | 58.43 (13.36–155.39) | 16.03 (-34.79–102.43) |
| CJ professionals | 180.97 (89.92–306.96) | 162.71 (82.96–262.16) | -18.26 (-136.65–86.57) |
| Victim of crime | 1,910.40 (903.60–3,342.86) | 1,405.42 (674.22–2,304.55) | -504.98 (-1,805.46–620.67) |
| Crime committed | 545.47 (147.90–1,154.34) | 743.12 (150.96–1,549.38) | 197.64 (-421.56–964.63) |
| Addiction keyworker | 237.15 (174.87–335.61) | 183.77 (145.13–232.79) | -53.38 (-146.56–11.17) |
| Trial medication | 741.49 (670.96–812.44) | 1,406.20 (1,325.56–1,482.39) | 664.71 (559.00–769.60) |
| Concomitant medication | 1,439.91 (289.00–2,936.04) | 1,914.42 (714.02–3,520.35) | 474.51 (-1,447.81–2,420.18) |
| Total | 7,171.45 (5,002.06–9,930.88) | 7,503.48 (5,630.40–9,808.68) | 332.03 (-2,691.61–3,292.67) |

### 2.2.3 Utilities

The distribution of participants’ responses to the EQ-5D-5L questionnaire by randomised treatment group is presented in [Figure S2.2](#fig-eq5-response-plot). Based on the bootstrapped imputed data, baseline utilities were 0·74 (95% CR 0·70, 0·78) and 0·74 (95% CR 0·70, 0·78) for the standard care and BUP-XR groups respectively. In the adjusted analysis, standard care was associated with a QALY of 0·13 (95% CR 0·08, 0·17) and BUP-XR a QALY of 0·15 (95% CR 0·10, 0·19). This corresponds to an incremental QALY of 0·02 (95% CR 0·00, 0·05).

| 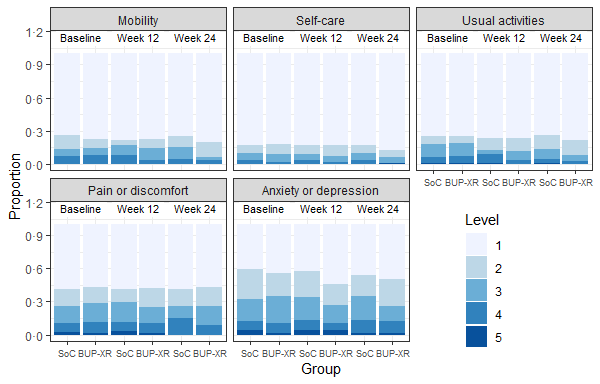  Figure S2.2: Distribution of participants’ responses to each EQ-5D attribute, by treatment allocated and time. Levels range from 1 to 5, with 5 representing the most severe problem. The proportion of completed responses. |
| --- |

A summary of utilities and QALYs based on responses to the EQ-5D-5L and EQ-VAS is presented in [Table S2.7](#tbl-eq5-response). In the standard care group, utilities remain approximately the same based on the EQ-5D-5L and show a general decrease based on EQ-VAS scores over the follow-up period. Both measures show a general increase in utilities for the BUP-XR group.

Table S2.7: Utilities based on responses to the EQ-5D-5L and EQ-VAS thermometer, by timepoint, and intervention group. Data are mean (SD).

| Group | Baseline | Week 12 | Week 24 | QALYs |
| --- | --- | --- | --- | --- |
| **EQ-5D-5L** | | | | |
| BUP-XR | 0·74 (0·26) | 0·77 (0·26) | 0·78 (0·23) | 0·36 (0·10) |
| SoC | 0·74 (0·27) | 0·73 (0·31) | 0·74 (0·28) | 0·34 (0·11) |
| **EQ-VAS** | | | | |
| BUP-XR | 0·63 (0·21) | 0·69 (0·20) | 0·69 (0·21) | 0·32 (0·07) |
| SoC | 0·65 (0·21) | 0·62 (0·22) | 0·61 (0·21) | 0·29 (0·07) |

### 2.2.4 Incremental analysis

#### 2.2.4.1 Base case analysis

Based on the point-estimate mean costs and QALYs, XR-BUP was more costly (£1,033) and more effective (0·02 QALYs) than standard care. The resulting ICER of £47,540 per QALY gained exceeds both the £20k and £30k per QALY WTP thresholds and BUP-XR is therefore associated with negative net monetary benefits at both thresholds ([Table S2.9](#tbl-icer-summary)). [Figure S2.3](#fig-societal-base-icer-scatter) shows that 80·09% of the bootstrapped results were in the NE quadrant, indicating that BUP-XR is associated with higher costs and more QALYs than standard care. Based on the bootstrapped results, it is unlikely that BUP-XR has lower costs (17·19% probability) and unlikely that BUP-XR results in fewer QALYs (3·30% probability).

In the primary analysis, at WTP thresholds of £20k and £30k per QALY, the probabilities that the intervention is cost effective from a societal perspective are 29·05% and 36·81%, respectively (see [Figure S2.4](#fig-inmb-ceac-base)).

| 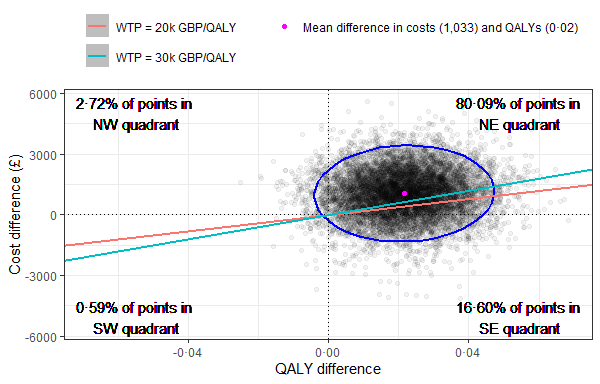  Figure S2.3: Cost-effectiveness plane showing the bootstrapped mean differences and 95% confidence ellipse in imputed total adjusted costs and QALYs of BUP-XR compared to standard care. |
| --- |
| 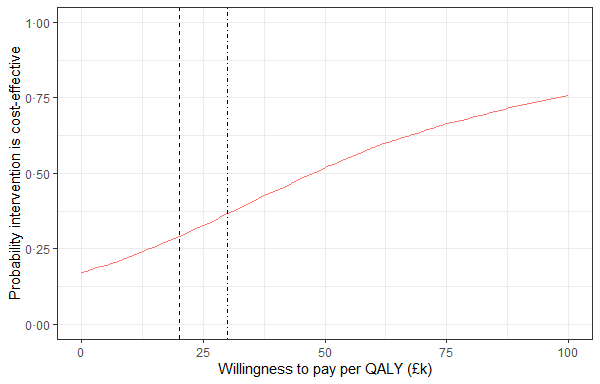  Figure S2.4: Cost-effectiveness acceptability curve for base case analysis. Dashed line represents £20k per QALY WTP. Dashed and dotted line represents £30k per QALY WTP. |

#### 2.2.4.2 Univariate sensitivity analyses

The results of the univariate sensitivity analyses presented in [Figure S2.5](#fig-base-case-univariate-sensitivity) indicated that there was no single cost input that, when varied by ±10% would have changed the cost-effectiveness decision at the £30k per QALY threshold. The cost of trial medication had the greatest impact on INMB, followed by concomitant medication, accommodation, and criminal justice costs, with differences in INMB when varied by ±10% of 166, 106, 52, and 29 respectively. Differences in INMBs in the remaining cost inputs were considerably smaller, with outpatient costs having the smallest difference (1·54).

| 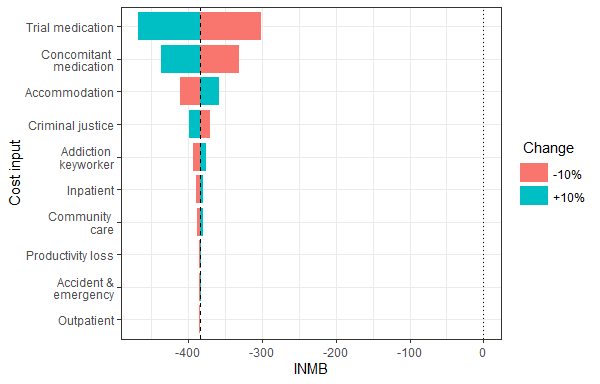  Figure S2.5: Tornado plot of univariate sensitivity analyses. Incremental NMB at a WTP of £30k per QALY when each cost input is varied by ±10%, ordered by decreasing order of impact on the INMB. |
| --- |

#### 2.2.4.3 QALY based on EQ-VAS score

Based on the point-estimate adjusted mean costs and QALYs derived from the EQ-VAS, XR-BUP was more costly and more effective than standard care. The resulting ICER of £40,310 per QALY gained exceeds both the £20k and £30k per QALY WTP thresholds and BUP-XR remains associated with negative monetary benefits at both thresholds (see [Table S2.9](#tbl-icer-summary)).

#### 2.2.4.4 Scenario analyses

Scenario analyses conducted from a societal perspective show that there is considerable variation in ICERs depending on the assumptions for cost inputs (ranging from £26,546 to £66,383 per QALY gained). The only scenario analysis with an ICER lower than £30k per QALY was the scenario where oral buprenorphine costs and oral buprenorphine/methadone dispensing costs were high (i.e., February 2019 prices with six supervised pick-ups per week for all standard care patients). In all other scenarios, the ICER was appreciably higher than the £20k and £30k per QALY WTP thresholds.

Table S2.7: Summary of cost-utility analyses based on bootstrapped and imputed data, adjusted for baseline costs and utilities. Data are mean (95% CR).

| Perspective | Scenario | Cost difference | QALY difference | INMB £20k/QALY | INMB £30k/QALY | ICER (£/QALY gained) |
| --- | --- | --- | --- | --- | --- | --- |
| Societal | As per base case | 1,033  (-1,189–3,225) | 0·02  (0·00–0·05) | -598  (-2,832–1,681) | -381  (-2,663–1,976) | 47,540 |
| Societal | VAS | 1,114  (-1,090–3,286) | 0·03  (0·01–0·05) | -561  (-2,716–1,654) | -285  (-2,462–1,949) | 40,310 |
| Societal | PSSRU Methadone | 859  (-1,761–3,033) | 0·02  (0·00–0·05) | -420  (-2,627–2,223) | -201  (-2,428–2,481) | 39,167 |
| Societal | Lower BUP-XR cost (£239·70) | 896  (-1,305–2,981) | 0·02  (0·00–0·05) | -462  (-2,608–1,738) | -246  (-2,475–1,965) | 41,324 |
| Societal | High oral buprenorphine/methadone dispensing costs | 839  (-1,375–2,959) | 0·02  (0·00–0·05) | -406  (-2,572–1,805) | -190  (-2,417–2,098) | 38,781 |
| Societal | Low oral buprenorphine/methadone dispensing costs | 1,155  (-1,018–3,332) | 0·02  (0·00–0·05) | -720  (-2,960–1,539) | -503  (-2,794–1,795) | 53,111 |
| Societal | High oral buprenorphine costs and high oral buprenorphine/methadone dispensing costs | 573  (-1,703–2,595) | 0·02  (0·00–0·05) | -141  (-2,234–2,094) | 75  (-2,097–2,344) | 26,546 |
| Societal | Low oral buprenorphine costs and low oral buprenorphine/methadone dispensing costs | 1,436  ( -792–3,660) | 0·02  (0·00–0·05) | -1,003  (-3,212–1,253) | -787  (-3,088–1,511) | 66,383 |
| NHS + PSS + CJ + Accom | As per base case | 1,001  (-1,209–3,186) | 0·02  (0·00–0·04) | -576  (-2,793–1,698) | -363  (-2,596–1,977) | 47,047 |
| NHS + PSS + CJ | As per base case | 1,300  ( -659–3,377) | 0·02  (0·00–0·04) | -885  (-3,043–1,089) | -678  (-2,892–1,330) | 62,717 |
| NHS + PSS | As per base case | 944  ( -136–2,264) | 0·02  (0·00–0·04) | -522  (-1,895–607) | -312  (-1,701–906) | 44,785 |
| NHS + PSS | Lower BUP-XR cost (£239·70) | 786  ( -275–2,018) | 0·02  (0·00–0·04) | -365  (-1,636–761) | -154  (-1,503–1,053) | 37,323 |
| NHS | As per base case | 962  ( -93–2,286) | 0·02  (0·00–0·04) | -534  (-1,879–589) | -320  (-1,764–868) | 44,955 |

The cost-effectiveness acceptability curves in [Figure S2.6](#fig-inmb-ceac) show the probabilities of BUP-XR being cost-effective, under each of the scenarios considered above, across a range of WTP thresholds.

When adopting an NHS + PSS cost perspective, the probabilities that the intervention is cost effective are 20·41% and 32·60% at the £20k and £30k per QALY thresholds, respectively. When the base case is adjusted such that the unit cost of BUP-XR is reduced by ~10.5% to £237·90, these probabilities increase to 33·19% and 41·08%, respectively from a societal perspective, and 27·71% and 41·08% from an NHS + PSS perspective.

| \| \| 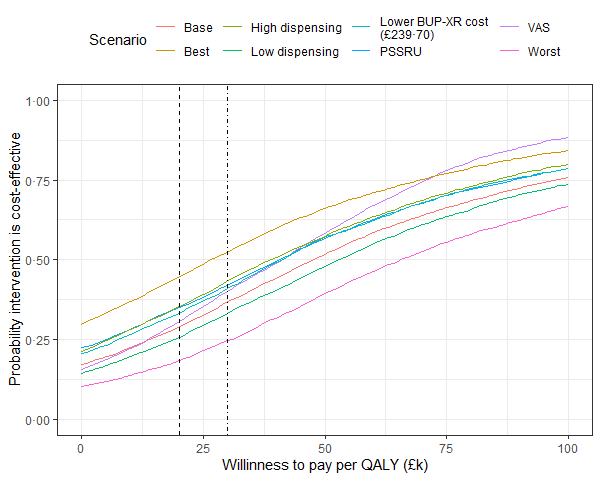  (a) Societal cost perspective scenarios \| \| --- \| \| \| --- \| --- \|  \| \| 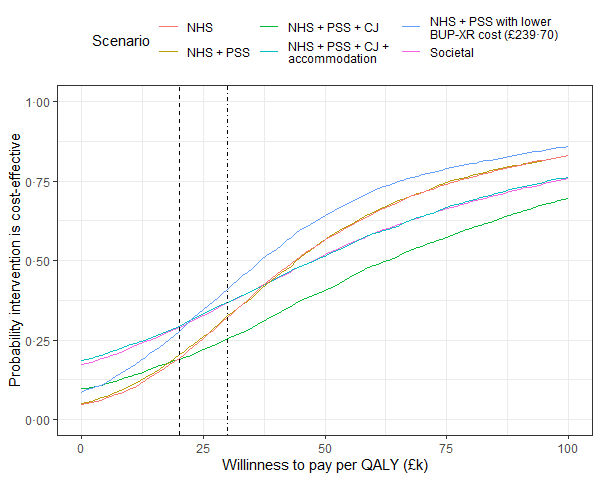  (b) Base case assumptions (unless specified), various cost perspectives \| \| --- \| \| \| --- \| --- \|   Figure S2.6: Cost-effectiveness acceptability curves for each analysis. Dashed line represents £20k per QALY WTP. Dashed and dotted line represents £30k per QALY WTP. |
| --- | --- | --- | --- | --- |

#### 2.2.4.4 Subgroup analyses

The results of the subgroup analysis suggest that BUP-XR is dominant when compared to standard care in patients who either: are rated as “severe” or higher on the CGI-S or whose current episode has surpassed 4 weeks.

Table S2.9: Results of subgroup analyses, based on bootstrapped and imputed data, adjusted for baseline costs and utilities, site, and injecting status. Data are mean (95% CR).

| Subgroup | Incremental costs (£) | Incremental QALYs | INMB @ 20k | INMB @ 30k | ICER |
| --- | --- | --- | --- | --- | --- |
| Benzodiazepine use | 3,499·67  (-5,588·20–13,181·19) | 0·03  (-0·03–0·11) | -2,853·71  (-12,430·74–6,483·08) | -2,530·73  (-12,215·92–7,004·67) | 108,356 |
| CGI severe | -3,147·17  (-11,435·23–4,149·28) | 0·05  (-0·01–0·11) | 4,135·96  (-3,261·84–12,391·22) | 4,630·35  (-2,875·76–12,981·96) | Dominant |
| Cocaine use | 3,062·34  (-3,552·56–10,435·21) | 0·02  (-0·03–0·08) | -2,623·20  (-10,022·54–4,043·81) | -2,403·63  (-9,821·41–4,407·43) | 139,469 |
| Current episode > 28 days | -102·46  (-4,367·89–3,369·50) | 0·02  (-0·01–0·05) | 575·68  (-2,925·52–4,850·37) | 812·29  (-2,779·29–5,088·91) | Dominant |

## 2.3 Discussion

The results of the cost-utility analysis suggest that using BUP-XR to treat opioid-use disorder is more costly than standard care oral medication and is associated with higher QALY gains. However, under base case assumptions, regardless of the perspective taken, BUP-XR was not cost-effective when compared to SoC at the WTP thresholds of £20k and £30k per QALY usually used by NICE in technology appraisals.^19^ This finding was robust in all except one of the sensitivity and scenario analyses, in the scenario where both oral medication acquisition and dispensing costs were high for all standard care participants the ICER for BUP-XR was <£30k per QALY gained. When considering patient subgroups, BUP-XR dominated SoC for patients who were either rated “Moderate-Extremely severe” on the CGI-S or whose current episode has lasted more than 28 days.

The results show that there is considerable uncertainty in the estimates of cost-effectiveness. Indeed, within the scenarios conducted from a societal cost perspective, the range of ICERs was ~£40,000 per QALY gained.

Based on the unadjusted imputed data and the univariate sensitivity analyses, the higher costs in the BUP-XR group are driven largely by the higher trial and concomitant medication costs. Previous studies of treatments for opioid-use disorder have suggested that their economic benefits result from reduced crime and victim of crime costs.^4,5,37^ In the present study, the BUP-XR group was associated with lower overall criminal justice costs than the SoC group during the trial follow-up — however, these differences were not significant. Somewhat counter-intuitively, the scenario analyses suggest that including criminal justice costs reduce the probability of BUP-XR being cost-effective. This is due to imbalances in costs at baseline, where mean total observed costs for criminal justice were ~£310 lower for the BUP-XR group than the SoC group.

The incremental QALYs are relatively small,^38^ however, this must be considered in the context of the eligible patient population. The EXPO inclusion criteria stated that patients must already be enrolled in either oral methadone or sublingual buprenorphine treatment and the baseline utility for EXPO participants was relatively high when compared to participants of other studies that also used the EQ-5D to measure utility in patients with opioid-use disorder^4,5^ although still lower than the age matched means in England.^39^ This may suggest that the EXPO participants were responding well to oral treatment and therefore had less room for improvement in utility than participants in previous trials that have found the intervention to be cost-effective. When considered alongside the subgroup analyses that show the treatment to be dominant for those rated as Moderate-Extremely-severe on the CGI, who also have a lower baseline utility (ΔM=0·08, 95% CI [0·02,0·14], t(263·60)=2·55, p=0·011), this *post-hoc* hypothesis is plausible and parsimonious.

There were three main limitations to the economic analysis of the EXPO trial: the precision of the resource use measures, the levels of missing data in the SoC group (due to patient drop-out), and the length of follow-up period. The imprecision of the resource use measures allowed a range of costs to be considered under the societal perspective but was nonetheless a substantive source of uncertainty. Whilst our statistical methods accounted for this, the linkage of trial data with routinely collected data for health costs (e.g., using Hospital Episode Statistics [HES] data) and criminal justice costs (e.g., Police National Computer [PNC] data), would reduce parameter uncertainty and limit the potential impact of recall bias.

The high-proportion of missing data in the SoC group due to trial drop-out adds to the uncertainty in the results, relying on the imputed data, and limited the methods for imputing data within the bootstrapped analysis. Including more variables makes the assumption that data is missing at random more plausible and violating this assumption would be a greater threat to the robustness of results than is posed by including trial group as a covariate instead of imputing by group.^26^

The 24-week trial period is similar to that in other randomised control trials for opioid-use disorder treatments.^4,5,40^ However, this may introduce time-horizon bias if the costs and effects are not realised within this period or extend beyond it. This may be a source of bias in the present study as the utility trajectory is different between the two treatment groups (i.e., utility increased over time in the BUP-XR group but reduced over time for the SoC group).

The EXPO trial is the first to compare an extended-release form of injectable buprenorphine with existing treatments for opioid-use disorder and collect data for a health economic analysis alongside clinical-effectiveness. Consequently, there is little empirical evidence regarding the cost-effectiveness of extended-release forms of injectable buprenorphine. There has been one previous direct comparison of an injectable buprenorphine product with oral buprenorphine/naloxone, however, this trial did not collect cost or quality of life data.^40^ In addition, the trial did not mirror UK practice.^41^

Buvidal®, another extended-release injectable buprenorphine product has been assessed in the UK by the All Wales Medicines Strategy Group and the Scottish Medicines Consortium. It is approved in Wales as a treatment option for opioid dependence.^42^ However, in the health technology assessment, cost-savings were assumed based on clinical expert opinion in the absence of published evidence and the comparator was oral buprenorphine/naloxone, which as shown above is more expensive than buprenorphine [Figure S2.1](#fig-bup-price-changes). Further, the assessment notes that utilities were derived from published literature and may “introduce bias of an unknown extent.”^42^

A similar assessment in Scotland approved Buvidal® for use in patients for whom oral methadone is not suitable.^43^ This assessment was based on a Markov-model based analysis, with a one-year time horizon. Utilities were not considered in their analysis (i.e., equal outcomes were assumed); costs were limited to medicine acquisition, administration/pharmacy, and other health resource use; and buprenorphine/naloxone was used as the comparator.^43^ In the base case, Buvidal® was considered cost-saving (-£140), however, changing the comparator from buprenorphine/naloxone to buprenorphine reversed this, with an incremental cost of £213.

The results presented in this report based on the analysis of data from the EXPO trial suggest that BUP-XR is not cost-effective at the indicative price, as a maintenance therapy for patients with opioid-use disorder unless they are rated moderate or higher on the CGI-S or their current episode has lasted more than 28 days.

## 2.4 Package citations

We used R^Version 4.3.1; 44^ and the R-packages *Amelia*^Version 1.8.1; 45^, *broom*^Version 1.0.5; 46,47^, *broom.mixed*^Version 0.2.9.4; 47^, *car*^Version 3.1.2; 48,49^, *carData*^Version 3.0.5; 49^, *coxed*^Version 0.3.3; 50^, *dplyr*^Version 1.1.2; 51^, *eq5d*^Version 0.14.0; 52^, *forcats*^Version 1.0.0; 53^, *furrr*^Version 0.3.1; 54^, *ggplot2*^Version 3.4.2; 55^, *haven*^Version 2.5.2; 56^, *Hmisc*^Version 5.1.0; 57^, *janitor*^Version 2.2.0; 58^, *kableExtra*^Version 1.3.4; 59^, *knitr*^Version 1.43; 60^, *lme4*^Version 1.1.33; 61^, *lmtest*^Version 0.9.40; 62^, *lubridate*^Version 1.9.2; 63^, *Matrix*^Version 1.5.4.1; 64^, *mgcv*^Version 1.8.42; 65,66–68^, *mice*^Version 3.16.0; 69^, *MKinfer*^Version 1.1; 70^, *nlme*^Version 3.1.162; 71^, *purrr*^Version 1.0.1; 72^, *Rcpp*^Version 1.0.10; 73,74^, *readr*^Version 2.1.4; 75^, *readxl*^Version 1.4.2; 76^, *RJ-2021-048*^77^, *rms*^Version 6.7.0; 78^, *stringr*^Version 1.5.0; 79^, *survival*^Version 3.5.5; 80^, *systemfit*^Version 1.1.30; 81^, *tibble*^Version 3.2.1; 82^, *tidyr*^Version 1.3.0; 83^, *tidyverse*^Version 2.0.0; 84^, *writexl*^Version 1.4.2; 85^, and *zoo*^Version 1.8.12; 86^ for our analyses.

## 2.5 References

1 Hardy WAS, Hughes DA. HEAP EXPO v1.0 20.07.2022 signed.pdf. Centre for Health Economics and Medicines Evaluation, Bangor University, 2022.

2 Husereau D, Drummond M, Augustovski F, *et al.* [Consolidated Health Economic Evaluation Reporting Standards (CHEERS) 2022 Explanation and Elaboration: A Report of the ISPOR CHEERS II Good Practices Task Force](https://doi.org/10.1016/J.JVAL.2021.10.008). *Value in Health* 2022; **25**: 10–31.

3 Barrett B, Byford S, Crawford MJ, *et al.* [Cost-effectiveness of screening and referral to an alcohol health worker in alcohol misusing patients attending an accident and emergency department: A decision-making approach](https://doi.org/10.1016/j.drugalcdep.2005.05.015). *Drug and Alcohol Dependence* 2006; **81**: 47–54.

4 Byford S, Barrett B, Metrebian N, *et al.* [Cost-effectiveness of injectable opioid treatment v. Oral methadone for chronic heroin addiction](https://doi.org/10.1192/bjp.bp.112.111583). *The British Journal of Psychiatry* 2013; **203**: 341–9.

5 Marsden J, Stillwell G, James K, *et al.* [Efficacy and cost-effectiveness of an adjunctive personalised psychosocial intervention in treatment-resistant maintenance opioid agonist therapy: A pragmatic, open-label, randomised controlled trial](https://doi.org/10.1016/S2215-0366(19)30097-5). *The Lancet Psychiatry* 2019; **6**: 391–402.

6 Lomas J, Asaria M, Bojke L, Gale CP, Richardson G, Walker S. [Which Costs Matter? Costs Included in Economic Evaluation and their Impact on Decision Uncertainty for Stable Coronary Artery Disease](https://doi.org/10.1007/s41669-018-0068-1). *PharmacoEconomics - Open* 2018; **2**: 403–13.

7 Indivior Inc. Sublocade(R) Prescribing Information. North Chesterfield, VA, 2022.

8 Buprenorphine | Drugs | BNF content published by NICE.

9 Joint Formulary Committee. British National Formulary. 2021.

10 Jones K, Burns A. Unit Costs of Health and Social Care 2021. Canterbury: Personal Social Services Research Unit, University of Kent, 2021.

11 NHS England. National Cost Collection for the NHS. 2021.

12 Brookes N, Barrett B, Netten A, Knapp E. Unit Costs in Criminal Justice (UCCJ). 2013.

13 Heeks M, Reed S, Tafsiri M, Prince S, 2nd. The economic and social costs of crime. London: Home Office, 2018.

14 Dubourg R, Hamed J, Thorns J. The economic and social costs of crime against individuals and households 2003/04. London: Home Office, 2005.

15 Brand S, Price R. The economic and social costs of crime. London: Home Office, 2000.

16 Office for National Statistics. Improving estimates of repeat victimisation derived from the Crime Survey for England and Wales. 2017; published online Oct.

17 Drummond M. Methods for the Economic Evaluation of Health Care Programmes. Oxford: Oxford University Press, 2005.

18 Office for National Statistics. Annual Survey of Hours and Earnings time series of selected estimates - Office for National Statistics. 2021; published online Oct.

19 National Institute for Health and Care Excellence. NICE health technology evaluations: The manual. 2022.

20 Pharmacy Services Negotiating Committee. Fees and allowances - PSNC Website. 2022.

21 Devlin N, Shah K, Feng Y, Mulhern B, van Hout B. [Valuing health-related quality of life: An EQ-5D-5L value set for England](https://doi.org/10.1002/hec.3564). *Health Economics* 2017; **27**: 7–22.

22 Hernandez Alava M, Wailoo A, Pudney S. Methods for mapping between the EQ-5D-5L and the 3L for technology appraisal. Sheffield: NICE Decision Support Unit, 2017.

23 Leurent B, Gomes M, Faria R, Morris S, Grieve R, Carpenter JR. [Sensitivity Analysis for Not-at-Random Missing Data in Trial-Based Cost-Effectiveness Analysis: A Tutorial](https://doi.org/10.1007/s40273-018-0650-5). *PharmacoEconomics* 2018; **36**: 889–901.

24 Simons CL, Rivero-Arias O, Yu L-M, Simon J. [Multiple imputation to deal with missing EQ-5D-3L data: Should we impute individual domains or the actual index?](https://doi.org/10.1007/s11136-014-0837-y) *Quality of Life Research* 2015; **24**: 805–15.

25 van Buuren S. Flexible Imputation of Missing Data, Second. Chapman & Hall, 2018.

26 Sullivan TR, White IR, Salter AB, Ryan P, Lee KJ. [Should multiple imputation be the method of choice for handling missing data in randomized trials?](https://doi.org/10.1177/0962280216683570) *Statistical Methods in Medical Research* 2018; **27**: 2610–26.

27 Brand J, Van Buuren S, Le Cessie S, Van Den Hout W. [Combining multiple imputation and bootstrap in the analysis of cost-effectiveness trial data](https://doi.org/10.1002/sim.7956). *Statistics in Medicine* 2018; **38**: 210–20.

28 Ramsey SD, Willke RJ, Glick H, *et al.* [Cost-Effectiveness Analysis Alongside Clinical Trials II](https://doi.org/10.1016/j.jval.2015.02.001). *Value in Health* 2015; **18**: 161–72.

29 Manca A, Hawkins N, Sculpher MJ. [Estimating mean QALYs in trial-based cost-effectiveness analysis: The importance of controlling for baseline utility](https://doi.org/10.1002/HEC.944). *Health Economics* 2005; **14**: 487–96.

30 Barber J, Thompson S. [Multiple regression of cost data: Use of generalised linear models](https://doi.org/10.1258/1355819042250249). *Journal of Health Services Research & Policy* 2004; **9**: 197–204.

31 Dodd S, Bassi A, Bodger K, Williamson P. [A comparison of multivariable regression models to analyse cost data](https://doi.org/10.1111/j.1365-2753.2006.00610.x). *Journal of Evaluation in Clinical Practice* 2006; **12**: 76–86.

32 Islam N, Cole TJ, Ross JS, Feeney T, Loder E. [Post-submission changes to prespecified statistical analysis plans](https://doi.org/10.1136/bmj.o2244). *BMJ* 2022; **378**: o2244.

33 Fenwick E, Claxton K, Sculpher M. [Representing uncertainty: The role of cost-effectiveness acceptability curves](https://doi.org/10.1002/hec.635). *Health Economics* 2001; **10**: 779–87.

34 Clinical Guidelines on Drug Misuse and Dependence Update 2017, Independent Expert Working Group, Independent Expert Working Group. Drug misuse and dependence: UK guidelines on clinical management. London: Department of Health, 2017.

35 Hoch JS, Briggs AH, Willan AR. [Something old, something new, something borrowed, something blue: A framework for the marriage of health econometrics and cost-effectiveness analysis](https://doi.org/10.1002/hec.678). *Health Economics* 2002; **11**: 415–30.

36 Sculpher M. [Subgroups and Heterogeneity in Cost-Effectiveness Analysis](https://doi.org/10.2165/00019053-200826090-00009). *PharmacoEconomics* 2008; **26**: 799–806.

37 Russolillo A, Moniruzzaman A, McCandless LC, Patterson M, Somers JM. [Associations between methadone maintenance treatment and crime: A 17-year longitudinal cohort study of Canadian provincial offenders](https://doi.org/10.1111/add.14059). *Addiction* 2018; **113**: 656–67.

38 Polak TB, Cucchi DG, Darrow JJ, Versteegh MM. [Incremental benefits of novel pharmaceuticals in the UK: A cross-sectional analysis of NICE technology appraisals from 2010 to 2020](https://doi.org/10.1136/bmjopen-2021-058279). *BMJ Open* 2022; **12**: e058279.

39 Feng Y, Devlin N, Herdman M. [Assessing the health of the general population in England: How do the three- and five-level versions of EQ-5D compare?](https://doi.org/10.1186/s12955-015-0356-8) *Health and Quality of Life Outcomes* 2015; **13**: 171.

40 Lofwall MR, Walsh SL, Nunes EV, *et al.* [Weekly and Monthly Subcutaneous Buprenorphine Depot Formulations vs Daily Sublingual Buprenorphine with Naloxone for Treatment of Opioid Use Disorder: A Randomized Clinical Trial](https://doi.org/10.1001/jamainternmed.2018.1052). *JAMA Internal Medicine* 2018; **178**: 764–73.

41 National Institute for Health and Clinical Excellence (NICE). Opioid dependence: Buprenorphine prolonged release injection (Buvidal). Evidence review. 2019.

42 All Wales Therapeutics and Toxicology Centre. WMSG Secretariat Assessment Report. Buprenorphine. Buprenorphine (Buvidal) 8 mg, 16 mg, 24 mg, 32 mg, 64 mg, 96 mg and 128 mg prolonged- release solution for subcutaneous injection. Reference number: 3977. 2019.

43 Scottish Medicines Consortium. Buprenorphine 8/16/24/32/64/96/128 mg prolonged-release solution for injection (Buvidal) SMC2169. 2019.

44 R Core Team. R: A language and environment for statistical computing. Vienna, Austria: R Foundation for Statistical Computing, 2023 <https://www.R-project.org/>.

45 Honaker J, King G, Blackwell M. [Amelia II: A program for missing data](https://doi.org/10.18637/jss.v045.i07). *Journal of Statistical Software* 2011; **45**: 1–47.

46 Robinson D, Hayes A, Couch S. Broom: Convert statistical objects into tidy tibbles. 2023 <https://CRAN.R-project.org/package=broom>.

47 Bolker B, Robinson D. Broom.mixed: Tidying methods for mixed models. 2022 <https://CRAN.R-project.org/package=broom.mixed>.

48 Fox J, Weisberg S. An R companion to applied regression, Third. Thousand Oaks CA: Sage, 2019 <https://socialsciences.mcmaster.ca/jfox/Books/Companion/>.

49 Fox J, Weisberg S, Price B. carData: Companion to applied regression data sets. 2022 <https://CRAN.R-project.org/package=carData>.

50 Kropko J, Harden JJ. Coxed: Duration-based quantities of interest for the cox proportional hazards model. 2020 <https://CRAN.R-project.org/package=coxed>.

51 Wickham H, François R, Henry L, Müller K, Vaughan D. Dplyr: A grammar of data manipulation. 2023 <https://CRAN.R-project.org/package=dplyr>.

52 Morton F, Nijjar JS. eq5d: Methods for analysing ’EQ-5D’ data and calculating ’EQ-5D’ index scores. 2023 <https://CRAN.R-project.org/package=eq5d>.

53 Wickham H. Forcats: Tools for working with categorical variables (factors). 2023 <https://CRAN.R-project.org/package=forcats>.

54 Vaughan D, Dancho M. Furrr: Apply mapping functions in parallel using futures. 2022 <https://CRAN.R-project.org/package=furrr>.

55 Wickham H. ggplot2: Elegant graphics for data analysis. Springer-Verlag New York, 2016 <https://ggplot2.tidyverse.org>.

56 Wickham H, Miller E, Smith D. Haven: Import and export ’SPSS’, ’stata’ and ’SAS’ files. 2023 <https://CRAN.R-project.org/package=haven>.

57 Harrell Jr FE. Hmisc: Harrell miscellaneous. 2023 <https://CRAN.R-project.org/package=Hmisc>.

58 Firke S. Janitor: Simple tools for examining and cleaning dirty data. 2023 <https://CRAN.R-project.org/package=janitor>.

59 Zhu H. kableExtra: Construct complex table with ’kable’ and pipe syntax. 2021 <https://CRAN.R-project.org/package=kableExtra>.

60 Xie Y. Dynamic documents with R and knitr, 2nd edn. Boca Raton, Florida: Chapman; Hall/CRC, 2015 <https://yihui.org/knitr/>.

61 Bates D, Mächler M, Bolker B, Walker S. [Fitting linear mixed-effects models using lme4](https://doi.org/10.18637/jss.v067.i01). *Journal of Statistical Software* 2015; **67**: 1–48.

62 Zeileis A, Hothorn T. [Diagnostic checking in regression relationships](https://CRAN.R-project.org/doc/Rnews/). *R News* 2002; **2**: 7–10.

63 Grolemund G, Wickham H. [Dates and times made easy with lubridate](https://www.jstatsoft.org/v40/i03/). *Journal of Statistical Software* 2011; **40**: 1–25.

64 Bates D, Maechler M, Jagan M. Matrix: Sparse and dense matrix classes and methods. 2023 <https://CRAN.R-project.org/package=Matrix>.

65 Wood SN. Fast stable restricted maximum likelihood and marginal likelihood estimation of semiparametric generalized linear models. *Journal of the Royal Statistical Society (B)* 2011; **73**: 3–36.

66 Wood SN, N., Pya, S"afken B. Smoothing parameter and model selection for general smooth models (with discussion). *Journal of the American Statistical Association* 2016; **111**: 1548–75.

67 Wood SN. Stable and efficient multiple smoothing parameter estimation for generalized additive models. *Journal of the American Statistical Association* 2004; **99**: 673–86.

68 Wood SN. Thin-plate regression splines. *Journal of the Royal Statistical Society (B)* 2003; **65**: 95–114.

69 van Buuren S, Groothuis-Oudshoorn K. [mice: Multivariate imputation by chained equations in r](https://doi.org/10.18637/jss.v045.i03). *Journal of Statistical Software* 2011; **45**: 1–67.

70 Kohl M. MKinfer: Inferential Statistics. 2023 <https://www.stamats.de>.

71 Pinheiro JC, Bates DM. Mixed-effects models in s and s-PLUS. New York: Springer, 2000 DOI:[10.1007/b98882](https://doi.org/10.1007/b98882).

72 Wickham H, Henry L. Purrr: Functional programming tools. 2023 <https://CRAN.R-project.org/package=purrr>.

73 Eddelbuettel D, François R. [Rcpp: Seamless R and C++ integration](https://doi.org/10.18637/jss.v040.i08). *Journal of Statistical Software* 2011; **40**: 1–18.

74 Eddelbuettel D, Balamuta JJ. [Extending extitR with extitC++: A Brief Introduction to extitRcpp](https://doi.org/10.1080/00031305.2017.1375990). *The American Statistician* 2018; **72**: 28–36.

75 Wickham H, Hester J, Bryan J. Readr: Read rectangular text data. 2023 <https://CRAN.R-project.org/package=readr>.

76 Wickham H, Bryan J. Readxl: Read excel files. 2023 <https://CRAN.R-project.org/package=readxl>.

77 Bengtsson H. [A unifying framework for parallel and distributed processing in r using futures](https://doi.org/10.32614/RJ-2021-048). *The R Journal* 2021; **13**: 208–27.

78 Harrell Jr FE. Rms: Regression modeling strategies. 2023 <https://CRAN.R-project.org/package=rms>.

79 Wickham H. Stringr: Simple, consistent wrappers for common string operations. 2022 <https://CRAN.R-project.org/package=stringr>.

80 Terry M. Therneau, Patricia M. Grambsch. Modeling survival data: Extending the Cox model. New York: Springer, 2000.

81 Henningsen A, Hamann JD. [Systemfit: A package for estimating systems of simultaneous equations in r](https://www.jstatsoft.org/v23/i04/). *Journal of Statistical Software* 2007; **23**: 1–40.

82 Müller K, Wickham H. Tibble: Simple data frames. 2023 <https://CRAN.R-project.org/package=tibble>.

83 Wickham H, Vaughan D, Girlich M. Tidyr: Tidy messy data. 2023 <https://CRAN.R-project.org/package=tidyr>.

84 Wickham H, Averick M, Bryan J, *et al.* [Welcome to the tidyverse](https://doi.org/10.21105/joss.01686). *Journal of Open Source Software* 2019; **4**: 1686.

85 Ooms J. Writexl: Export data frames to excel ’xlsx’ format. 2023 <https://CRAN.R-project.org/package=writexl>.

86 Zeileis A, Grothendieck G. [Zoo: S3 infrastructure for regular and irregular time series](https://doi.org/10.18637/jss.v014.i06). *Journal of Statistical Software* 2005; **14**: 1–27.

87 Connock M, Juarez-Garcia A, Jowett S, *et al.* Methadone and buprenorphine for the management of opioid dependence: A systematic review and economic evaluation. *Health Technology Assessment* 2007; **11**. DOI:[10.3310/hta11090](https://doi.org/10.3310/hta11090).

88 Department of Health. Reference costs 2015-2016. 2015.

89 Mills H, Skodbo S, Blyth P. Understanding organised crime: Estimating the scale and the social and economic costs Research Report 73. 2013.

90 National Institute for Health and Care Excellence. Non-alcoholic fatty liver disease (NAFLD): Assessment and management. 2016.

91 National Police Chiefs’ Council. National Policing Guidelines on Charging for Police Services. 2018.

92 Newton A, May X, Eames S, Ahmad M. Economic and social costs of reoffending. London: Ministry of Justice, 2019.

93 Nicholas Pleace. At what cost? An estimation of the financial costs of single homelessness in the UK. London: Crisis, 2015.

94 Third Sector. Analysis: Counting the cost of reform at Samaritans. 2013.

95 Walker N, Yang Y, Kiparoglou V, Pokhrel S, Robinson H, van Woerden H. [An examination of user costs in relation to smokers using a cessation service based in the UK](https://doi.org/10.1186/s12913-018-2985-1). *BMC Health Services Research* 2018; **18**: 182.
